# Distinct molecular profiles of skull bone marrow in health and neurological disorders

**DOI:** 10.1016/j.cell.2023.07.009

**Published:** 2023-08-17

**Authors:** Zeynep Ilgin Kolabas, Louis B. Kuemmerle, Robert Perneczky, Benjamin Förstera, Selin Ulukaya, Mayar Ali, Saketh Kapoor, Laura M. Bartos, Maren Büttner, Ozum Sehnaz Caliskan, Zhouyi Rong, Hongcheng Mai, Luciano Höher, Denise Jeridi, Muge Molbay, Igor Khalin, Ioannis K. Deligiannis, Moritz Negwer, Kenny Roberts, Alba Simats, Olga Carofiglio, Mihail I. Todorov, Izabela Horvath, Furkan Ozturk, Selina Hummel, Gloria Biechele, Artem Zatcepin, Marcus Unterrainer, Johannes Gnörich, Jay Roodselaar, Joshua Shrouder, Pardis Khosravani, Benjamin Tast, Lisa Richter, Laura Díaz-Marugán, Doris Kaltenecker, Laurin Lux, Ying Chen, Shan Zhao, Boris-Stephan Rauchmann, Michael Sterr, Ines Kunze, Karen Stanic, Vanessa W.Y. Kan, Simon Besson-Girard, Sabrina Katzdobler, Carla Palleis, Julia Schädler, Johannes C. Paetzold, Sabine Liebscher, Anja E. Hauser, Ozgun Gokce, Heiko Lickert, Hanno Steinke, Corinne Benakis, Christian Braun, Celia P. Martinez-Jimenez, Katharina Buerger, Nathalie L. Albert, Günter Höglinger, Johannes Levin, Christian Haass, Anna Kopczak, Martin Dichgans, Joachim Havla, Tania Kümpfel, Martin Kerschensteiner, Martina Schifferer, Mikael Simons, Arthur Liesz, Natalie Krahmer, Omer A. Bayraktar, Nicolai Franzmeier, Nikolaus Plesnila, Suheda Erener, Victor G. Puelles, Claire Delbridge, Harsharan Singh Bhatia, Farida Hellal, Markus Elsner, Ingo Bechmann, Benjamin Ondruschka, Matthias Brendel, Fabian J. Theis, Ali Erturk

**Affiliations:** 1 Institute for Tissue Engineering and Regenerative Medicine (iTERM), Helmholtz Center, Neuherberg, Munich, Germany; 2 Institute for Stroke and Dementia Research, LMU University Hospital, Ludwig-Maximilians University Munich, Munich, Germany; 3 Graduate School of Systemic Neurosciences (GSN), Munich, Germany; 4 Institute of Computational Biology, Helmholtz Zentrum München, German Research Center for Environmental Health, Neuherberg, Germany; 5 Division of Mental Health in Older Adults and Alzheimer Therapy and Research Center, Department of Psychiatry and Psychotherapy, University Hospital, Ludwig Maximilian University Munich, 80336 Munich, Germany; 6 German Center for Neurodegenerative Diseases (DZNE) Munich, Munich, Germany; 7 Ageing Epidemiology (AGE) Research Unit, School of Public Health, Imperial College London, London, UK; 8 Munich Cluster for Systems Neurology (SyNergy), Munich, Germany; 9 Sheffield Institute for Translational Neuroscience, University of Sheffield, Sheffield, UK; 10 Department of Nuclear Medicine, University Hospital, Ludwig-Maximilians-Universität München, Munich, Germany; 11 Institute for Diabetes and Obesity, Helmholtz Center Munich and German Center for Diabetes Research (DZD), 85764 Neuherberg, Germany; 12 Munich Medical Research School (MMRS), 80336 Munich, Germany; 13 Helmholtz Pioneer Campus (HPC), Helmholtz Munich, Neuherberg, Germany; 14 Wellcome Sanger Institute, Cambridge, UK; 15 School of Computation, Information and Technology (CIT), TUM, Boltzmannstr. 3, 85748 Garching, Germany; 16 Department of Radiology, University Hospital, LMU Munich, Munich, Germany; 17 Charité - Universitätsmedizin Berlin, Department of Rheumatology and Clinical Immunology, Berlin, Germany; 18 Immune Dynamics, Deutsches Rheuma-Forschungszentrum (DRFZ), a Leibniz Institute, Berlin, Germany; 19 Biomedical Center (BMC), Core Facility Flow Cytometry, Faculty of Medicine, LMU Munich, Munich, Germany; 20 Institute for Diabetes and Cancer, Helmholtz Munich, Munich, Germany; 21 Institute of Neuroradiology, University Hospital LMU, Munich, Germany; 22 Institute of Diabetes and Regeneration Research, Helmholtz Diabetes Center, Helmholtz Zentrum München, Neuherberg, Germany; 23 Institute of Stem Cell Research, Helmholtz Zentrum München, Neuherberg, Germany; 24 Institute of Clinical Neuroimmunology, University Hospital Munich, Ludwig-Maximilians University Munich, Munich, Germany; 25 Department of Neurology, Ludwig-Maximilians-Universität München, Munich, Germany; 26 Institute of Legal Medicine, University Medical Center Hamburg-Eppendorf, Hamburg, Germany; 27 Department of Computing, Imperial College London, London, UK; 28 Institute of Anatomy, University of Leipzig, 04109 Leipzig, Germany; 29 Institute of Legal Medicine, Faculty of Medicine, LMU Munich, Germany; 30 TUM School of Medicine, Technical University of Munich, Munich, Germany; 31 Metabolic Biochemistry, Biomedical Center (BMC), Faculty of Medicine, Ludwig-Maximilians-Universität München, Munich, Germany; 32 Biomedical Center (BMC), Medical Faculty, Ludwig-Maximilians Universität Munich, Munich, Germany; 33 III. Department of Medicine, University Medical Center Hamburg-Eppendorf, Hamburg, Germany; 34 Hamburg Center for Kidney Health (HCKH), University Medical Center Hamburg-Eppendorf, Hamburg, Germany; 35 Department of Clinical Medicine, Aarhus University, Aarhus, Denmark; 36 Department of Pathology, Aarhus University Hospital, Aarhus, Denmark; 37 Institute of Pathology, Department of Neuropathology, Technical University Munich, TUM School of Medicine, Munich, Germany; 38 Department of Mathematics, Technische Universität München, Garching bei München, Germany

**Keywords:** skull-brain connection, neuroinflammation, immune cell trafficking, DISCO clearing, 3D-imaging, scRNA-seq, proteomics, PET imaging, neurological disorders, non-invasive monitoring

## Abstract

The bone marrow in the skull is important for shaping immune responses in the brain and meninges, but its molecular makeup among bones and relevance in human diseases remain unclear. Here, we show that the mouse skull has the most distinct transcriptomic profile compared with other bones in states of health and injury, characterized by a late-stage neutrophil phenotype. In humans, proteome analysis reveals that the skull marrow is the most distinct, with differentially expressed neutrophil-related pathways and a unique synaptic protein signature. 3D imaging demonstrates the structural and cellular details of human skull-meninges connections (SMCs) compared with veins. Last, using translocator protein positron emission tomography (TSPO-PET) imaging, we show that the skull bone marrow reflects inflammatory brain responses with a disease-specific spatial distribution in patients with various neurological disorders. The unique molecular profile and anatomical and functional connections of the skull show its potential as a site for diagnosing, monitoring, and treating brain diseases.

## Highlights

• Bone marrow across the body display molecular heterogeneity in mice and humans
• Calvaria cells have a distinct profile that is relevant to brain pathologies
• Structural details of human skull-meninges connections are revealed
• TSPO-PET imaging of human skulls can be a proxy of neuroinflammation in the brain

## Graphical abstract

**Figure:**
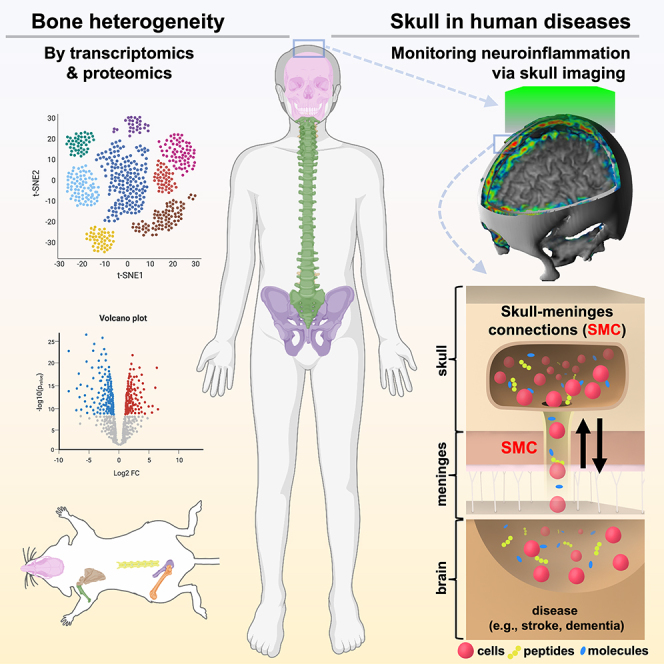

## Teaser

The bone marrow of the skull has a unique molecular and functional composition, as well as disease-specific inflammatory responses that can be observed through human translocator protein positron emission tomography (TSPO-PET) imaging, suggesting that it has potential as a site for brain disease diagnosis, monitoring, and treatment.

## Introduction

The complex interplay between immune cells at the central nervous system (CNS) borders and the CNS resident immune system has become the subject of intensive research.^1^ The dura mater of the meninges is directly connected to the adjacent skull bone marrow via skull-meninges connections (SMCs) that allow the trafficking of immune cells^2^^,^^3^^,^^4^^,^^5^ and might facilitate the preferential recruitment of immune cells to the meninges from the skull bone marrow.^5^^,^^6^^,^^7^

In mice, high-throughput, multidimensional techniques, such as flow and mass cytometry and single-cell RNA sequencing (scRNA-seq), have provided a detailed map of the cell-type composition and molecular profiles of meningeal immune cells.^8^^,^^9^^,^^10^^,^^11^^,^^12^ CNS border-derived cells can be functionally distinct from blood-derived cells of the same type^5^ and cells from different regions of the CNS borders.^12^^,^^13^

By contrast, little functional and multidimensional molecular data are available for the skull bone marrow and how it relates to other bones. For example, Herisson et al.^4^ found a higher influx of monocytes and neutrophils from the skull than from the tibia after brain injury, and Cugurra et al.^5^ showed that dural monocytes and neutrophils are mainly directly derived from the skull bone marrow. Basic scRNA-seq data of the unperturbed skull in comparison to the tibia marrow were obtained by Mazzitelli et al..^14^ Proteome-wide characterization of the bone marrow in mice has focused on individual cell types and bones in homeostasis^15^^,^^16^^,^^17^^,^^18^ or has used antibody-based methods.^19^ For the skull bone marrow, profiling has been limited to small flow or mass cytometry panels.^6^^,^^9^^,^^10^ Thus, it remains unclear whether the expression profiles of skull bone marrow cells are distinct from those of other bones and whether different types of bone marrows react differently to brain injury.

In humans, the functional roles and molecular makeups of the skull bone marrow, and other bone marrows are even less well characterized. A limited number of ‘omics studies of the human bone marrow have been presented,^20^^,^^21^^,^^22^^,^^23^ but a systematic characterization of potential differences among different bone marrows under different conditions is yet to be performed. Even on an anatomical level, although the presence of human SMCs has been suggested using microcomputed tomography (microCT),^4^ their detailed conformation at the cellular level remains elusive.

Here, we performed a systematic and comprehensive molecular analysis of the RNA and protein expression profiles of diverse bone marrow cells in mice and humans. In mice, we performed bulk and scRNA-seq and bulk proteomics on cells from six different bones, the dura, and the brain in three conditions (naive, middle cerebral artery occlusion [MCAo], and sham-operated animals). Our data show that different bones have distinct molecular profiles, with the skull calvaria bone displaying the highest number of differentially expressed genes (DEGs) and ligand-receptor (LR) pairs, mainly related to migration and inflammation.

For studies in humans, we collected post-mortem samples from the skull, vertebra, and pelvis of 20 deceased individuals and performed proteomic analysis, again showing a unique molecular profile of the skull. Using optical clearing on human skull + meninges + brain specimens, we characterized the anatomical details of SMCs at the cellular level. Using functional imaging in patients, we found disease-specific increases in 18 kDa translocator protein (TSPO) positron emission tomography (PET) signal in different parts of the skull in numerous neurological diseases and a strong correlation between changes in the brain and skull TSPO-PET signal in patients with Alzheimer’s disease (AD) and stroke in longitudinal data. These data provide a critical link between the skull and neurological diseases in humans.

## Results

### Skull is a dynamic site that responds to stroke

**Figure 1:**
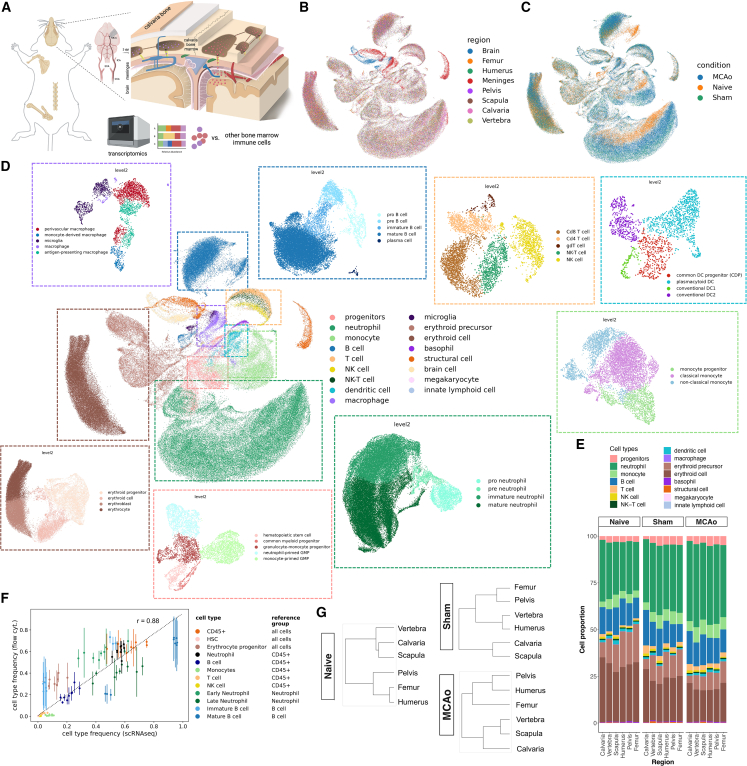
Bones diverge based on transcriptional signature of cell types (A) Experimental design of single-cell RNA sequencing of bones, dura, and brain, and a schematic of the middle cerebral artery occlusion (MCAo) model of stroke. (B–D) Uniform manifold approximation and projection (UMAP) distribution of scRNA-seq colored by (B) region, (C) condition: naive, sham-operated, and MCAo, and (D) cell type with fine annotated cell types in the surrounding with matching color. (E) Relative proportions of the coarse cell types. (F) Correlation between relative proportions of the cell types in scRNA-seq and independent animals measured by flow cytometry using 15 color panel. Mean Pearson correlation over conditions and bones is 0.875. (G) Dendrograms for naive, sham, and MCAo conditions. (n = 3 pooled animals for sham and n = 6 pooled animals for MCAo.). See also Figures S1 and S2.

To test the skull marrow’s involvement in the response to brain injury, we used MCAo as a model for stroke in mice.^24^ In MCAo, the mice first undergo a neck incision to expose the carotid artery before the occlusion of the middle cerebral artery (Figure 1A). A sham-operation procedure without MCAo mimics a local injury without inducing stroke.^24^^,^^25^^,^^26^

**Figure S1:**
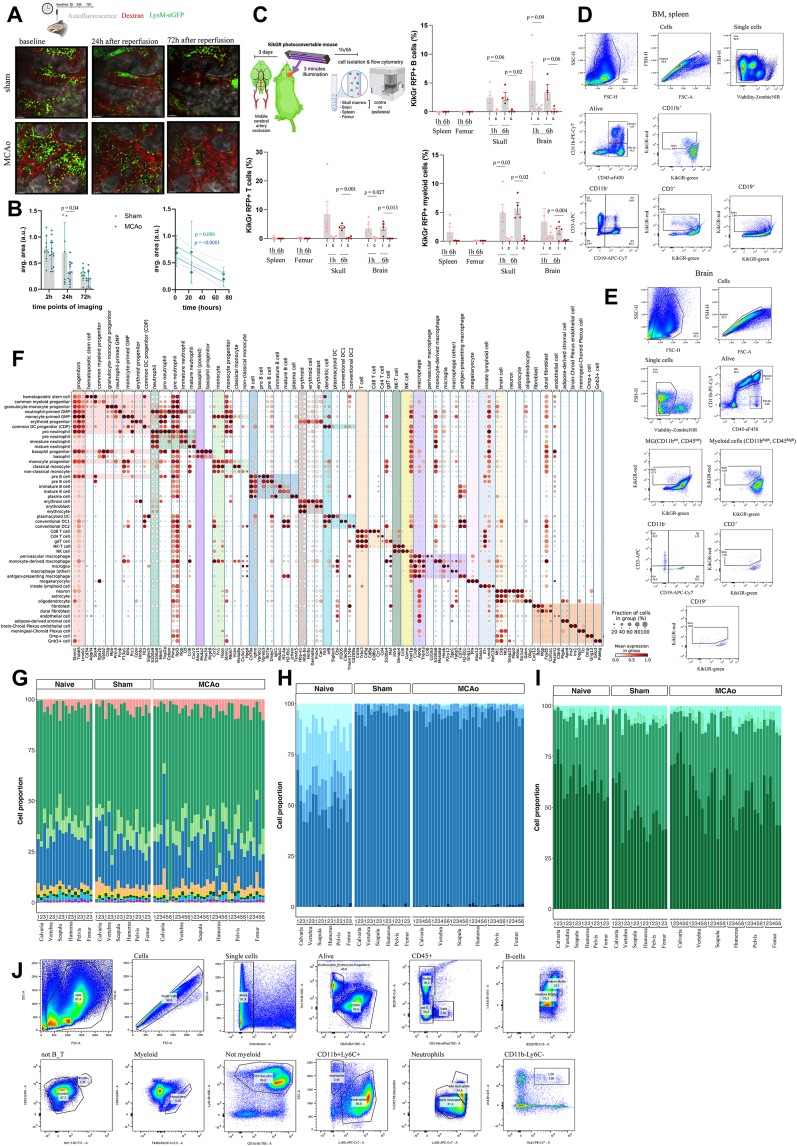
Assessment of skull cell dynamics and details of cell-type annotations, related to Figure 1 (A) Overview of the two-photon experiment and representative images from sham and MCAo groups. 2, 24, and 72 h after surgeries same ROIs were imaged. Per each imaging session, animals were given dextran for vessel labeling (n = 3 for naive and sham and n = 5 animals for MCAo). Scale bars, 50 μm. (B) Quantification of changes in area between sham and MCAo conditions. LysM was quantified based on maximum intensity projected time series of 3 frames per batch. Average area of LysM cells in MCAo is less than sham in 24 h (p = 0.04) and both conditions have significant decrease of LysM cells over time (p = 0.004 for sham and p <0.0001 in MCAo)Data represented as ±SEM.(see STAR Methods for details). (C) Photoconversion in KikGR mouse model to track cell trafficking from skull to brain 3 days after stroke. B cell (1 h, ipsi vs. contra skull, p = 0.06, brain, p = 0.09. 6 h ipsi vs. contra skull, p = 0.02, brain, p = 0.06), T cells (1 h, ipsi vs. contra brain, p = 0.027. 6 h ipsi vs. contra skull, p = 0.001, brain, p = 0.013), and myeloid cells (1 h, ipsi vs. contra skull, p = 0.03. 6 h ipsi vs. contra skull, p = 0.02, brain, p = 0.004) were analyzed within the skull and brain compartment at indicated time points. Data represented as ±SEM. (D and E) Gating strategy for B cells, T cells, myeloid cells in bone marrow and spleen (D) and in brain (E). (F) Coarse and fine annotated cell types and their marker genes. (G–I) Deconvolved pooled data using SNPs showing (G) coarse annotations, (H) B cell fine cell annotation, and (I) neutrophils fine cell annotations. (J) Gating strategy for proportions: B cells, T cells, monocytes, neutrophils, eosinophils, erythroid cells, progenitors, NK cells, late neutrophils, B cell progenitors for flow cytometry experiment demonstrating proportions.

Two-photon imaging on the skull after stroke (n = 3 for naive and sham, n = 5 for MCAo) at baseline and 2, 24, and 72 h post injury showed that both sham and MCAo groups had a significant decrease of LysM^+^ cells (mostly myeloid cells) (Figure S1A, p = 0.004 in sham and p ≤ 0.0001 in MCAo). Furthermore, there was a higher efflux of myeloid cells from the skull after stroke (Figure S1B), similar to what was observed for Ly6C^hi^ monocytes and neutrophils.^4^

Next, we studied immune cells in the skull marrow and brain using KikGR mouse model.^5^^,^^27^ We used ultraviolet laser illumination to convert a photoconvertible protein to RFP in the skull area above the ischemic brain region (Figures S1C–S1E). We detected RFP^+^ B, T, and myeloid cells in the ipsilateral brain 1 and 6 h after photoconversion (Figure S1C), indicating that immune cells from the skull marrow are recruited to the brain after injury.^2^^,^^4^^,^^5^^,^^6^

### Expression differences between cells of different bone marrows

Next, we assessed if/how skull cells might be different. To this end, we performed scRNA-seq analysis on three flat bones (calvaria, scapula, and pelvis [ilium]), two long bones (humerus and femur), and one irregular bone (vertebra from thoracic level T5 to lumbar L3), along with dura mater and brain samples in naive, sham-operated, and MCAo-operated animals (Figure 1A).

**Figure S2:**
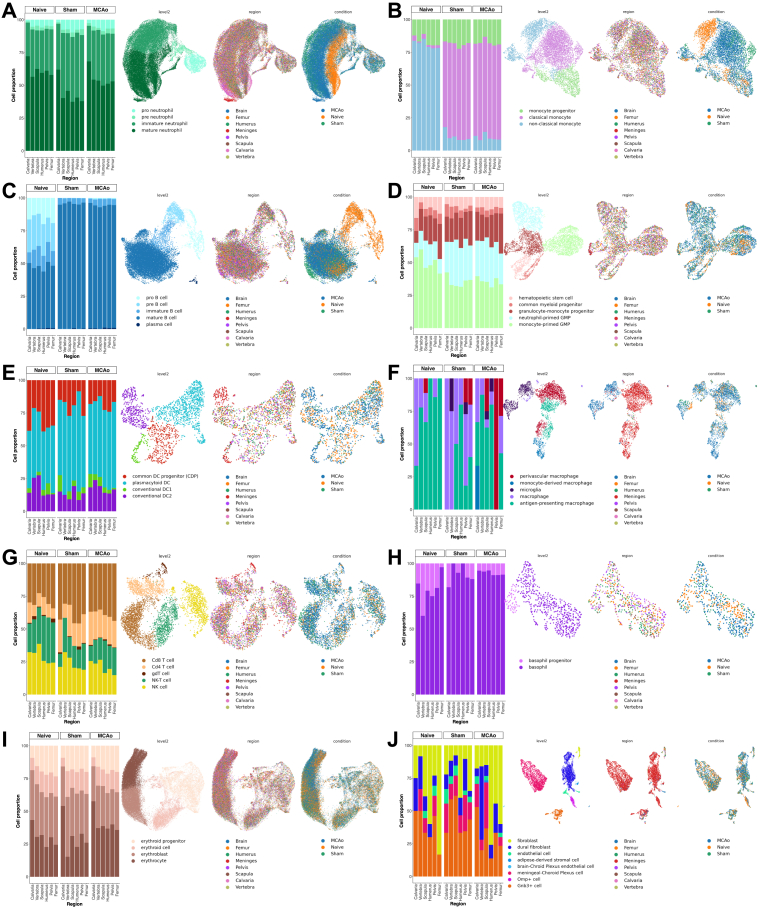
Proportions and UMAP of fine cell types over all conditions, related to Figure 1 Coarse cell types are shown separately with their fine cell-type proportion over three conditions, and their UMAP distribution for the cell-type, condition and region: (A) neutrophils, (B) monocytes, (C) B cells, (D) progenitors, (E) dendritic cells, (F) macrophages, (G) T and NK cells, (H) basophils, (I) erythroid cells, and (J) structural cells.

Single-cell transcriptomics of >100,000 cells across the bones and conditions revealed 17 coarse and 50 fine cell types (Figures 1B–1D). We found a bone-specific abundance of the coarse cell types, whereas meninges and brain-specific cells were separated (Figure 1B). We detected large numbers of neutrophils (∼25%) and erythroid cells (∼30%) along with other expected cell types (Figures 1E and S1F). Neutrophil populations were clearly separated between the conditions (Figure 1C). Standard cell-type proportions were homogeneously distributed among different bones (Figures 1E, 1F, S1G–S1J, and S2). Cell-type proportions were validated per mouse by deconvolving pooled samples with SNPs and flow cytometry, with an overall correlation of 0.88 (Figure 1F).

**Figure S3:**
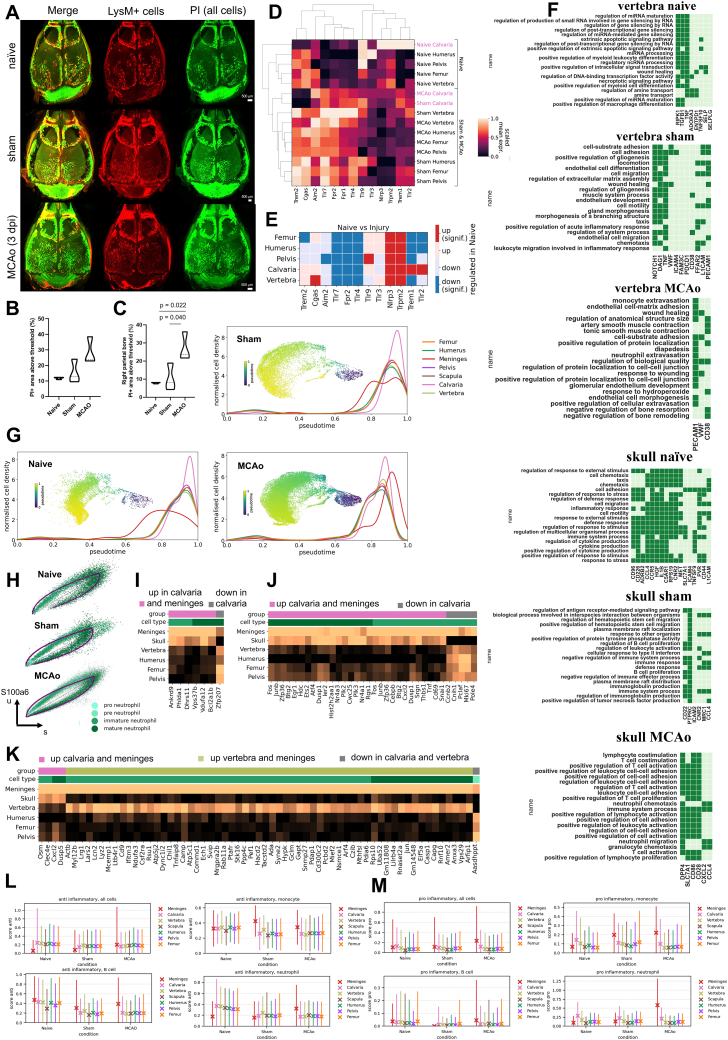
Analysis of skull cell numbers, neutrophil development, and inflammatory responses in different bones and the meninges, related to Figure 2 (A) Whole head clearing of LysM mice in naive, sham, and MCAo (stroke on left side) condition. (B) Quantification of PI signal in the frontal and parietal bones show a strong trend (F(2,6) = 5.027, p = 0.522) for increased PI signal in MCAo condition compared to sham (p = 0.124) and naive conditions (p = 0.053). (C) Quantification of PI signal in the contralateral parietal skull bone of show increase (F(2,6) = 8.323, p = 0.019) in PI signal in MCAo condition compared to sham (p = 0.040) and naive (p = 0.022) conditions (n = 3 per group); dpi, days post injury. (D) Expression of DAMP relevant genes in three conditions with their relative hierarchical clustering. (E) Comparison of naive vs. injury response of specific DAMP genes. Color code indicates significance (p < 0.05). (F) The unique LR pairs in the skull and vertebra in three different conditions. LR pairs that occur in at least 5 different cell-type pairs in a given bone group are shown. (permutation test, 1000 permutations, p = 0) (G) Pseudo-time analysis of naive, sham, and MCAo with normalized cell density in each condition for each region. (H) Phase portrait showing unspliced and spliced counts in neutrophils of gene *S100a6* for naive, sham and MCAo respectively. (I–K) Mean expressions of upregulated genes in meninges and in a single other group in (I) naive, (J) sham, and (K) MCAo. (L and M) Mean and standard deviation of (L) anti-inflammatory and (M) pro-inflammatory score over cells of all cell types, B cells, neutrophils, and monocytes in naive, sham, and MCAo (significance and LFC in Table S1, tabs 29 and 30). Inflammatory score is based on the expression of *Il6*, *Il1a*, *Il1b*, Ifng, Il11, Il7d, Il7f, Il18 and *Tnf* (pro-inflammatory) and *Il1rn*, *Tgfb1*, *Il4*, *Il10*, *Il12a*, and *Il13* (anti-inflammatory).

To investigate changes in absolute cell numbers, we imaged whole mouse bodies at cellular resolution using vDISCO tissue clearing^28^ and found that the number of total cells (propidium iodide [PI]-labeled cells) increased in the calvaria marrow of mice after stroke compared with controls (Figures S3A–S3C; Video S1). The overall increase in cell number contrasts with the decrease in LysM^+^ cells quantified by live imaging (Figure S1A), suggesting the mobilization of specific cell types out of the skull, whereas there is an overall increase in immune cell numbers as a response to injury.

**Figure 2:**
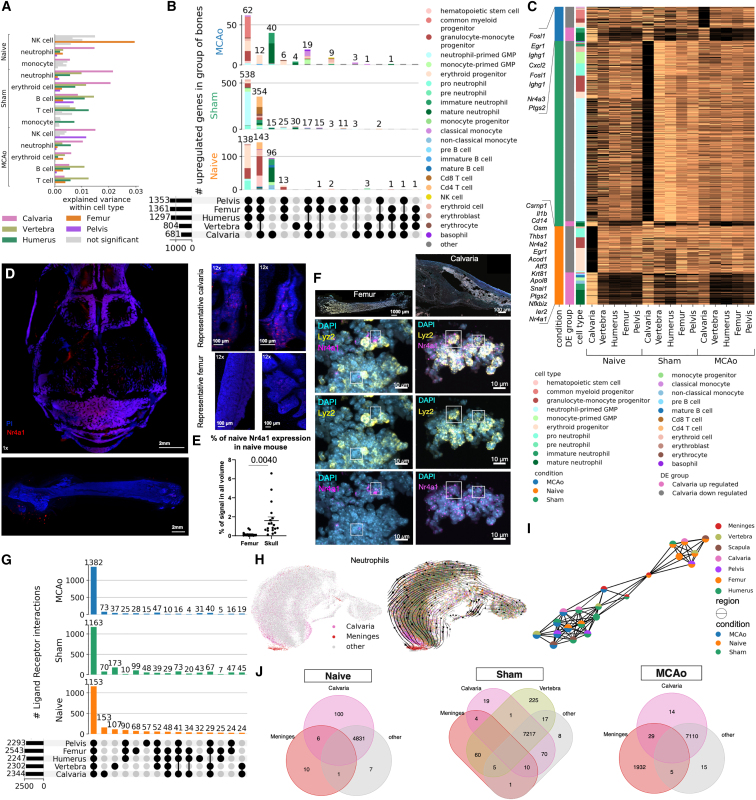
Different cell types show unique differentially expressed genes and ligand-receptor pairs between bones (A) PC regression plot shows how strongly each bone’s cell population diverges from the pooled population of other bones by variance explained for each coarse cell type. Only significant differences are shown for level 1 annotations. (permutation test, p < 0.0001) (B) Differentially expressed genes in naive, sham, and MCAo conditions (p < 0.05, LFC > 1 threshold). Each bar represents the fine cell-type color the genes are upregulated in. Fine cell annotations are used. (C) Calvaria-unique upregulated genes in the three conditions. (p < 0.05, LF change > 1) (D) Representative images of *Nr4a1* labeling after clearing and light-sheet fluorescent microscopy, n = 3. (E) Threshold based quantification of 12× scans of Nr4a1 (p = 0.0040). Nr4a1+ voxels as % of total volume. Data represented as ±SEM. (F) *Nr4a1* transcript is shown to colocalize with *Lyz2* and *Mpo*, myeloid cell marker and progenitor marker, respectively, using RNAscope. (G) Ligand-receptor interactions in three conditions on coarse cell-type annotations. (permutation test, 1000 permutations, p = 0) (H) Left: in the neutrophil subpopulation, calvaria, and dura neutrophils are highlighted in region-based UMAP. Right: projected developmental trajectory of MCAo neutrophils subset using scVelo. (I) PAGA analysis on the neutrophils subpopulation demonstrates separation of samples based on condition. (J) DE genes (DEGs) among dura, calvaria, and other bones, in three conditions (n = 3 pooled animals for sham and n = 6 pooled animals for MCAo). (p < 0.05, LF change > 0.5) See also Figures S3 and S4.

Video S1. Part (1) vDISCO whole-body cleared LysM mouse demonstrates the distribution of monocytes and neutrophils in the whole body after MCAo; part (2) vDISCO cleared LysM mouse head after MCAo has high cell intensity as well as LysM+ immune cells in the parietal and frontal skull, related to Figures 1 and 2

Hierarchical clustering showed that the long bones, femur, and humerus clustered together with the pelvis. Likewise, the two flat bones, scapula and calvaria, clustered together. The irregular vertebral bone branched with the flat bones in naive condition and after MCAo surgery and with the long bones and pelvis in sham condition (Figure 1G). Calvaria clustered with scapula in naive and sham conditions. Notably, it formed its own branch in MCAo condition, indicating a skull-specific immune reaction to brain injury.

To assess how strongly the gene expression profiles of one bone’s population diverge from the other bones’ pooled population for each cell type, we used principal component regression analysis (Figure 2A). The calvaria’s neutrophils diverged most from the neutrophils of the other bones in all three conditions.

On analyzing DEGs, we found the highest number in the calvaria for all conditions (Figure 2B; Table S1, tabs 5–13): in naive condition, 96; sham condition, 15; and MCAo condition, 62 genes were upregulated, whereas 138, 538, and 62 were downregulated, respectively. In all three conditions, most of the differentially downregulated genes in the calvaria were observed in progenitor cells such as pro-neutrophils, granulocyte-monocyte progenitors, and erythroid progenitors, whereas the upregulated genes were mostly in the myeloid lineage (Figure 2B).

In naive condition calvaria myeloid cell DEGs related to the regulation of apoptotic processes and programmed cell death pathways (Table S1, tabs 5–7), and calvaria-unique DEGs were mostly transcription factors, immediate early genes, and taxis-related genes (Figure 2C; Table S1, tabs 5–7). Transcription factors included *Nr4a1* and *Nr4a2* involved in cellular proliferation, apoptosis, metabolism, and T cell regulation,^29^ with an anti-inflammatory and damage-limiting role after ischemic stroke.^30^ Taxis-related DEGs include chemokines and chemokine receptors, e.g., *Cxcr4*, *Ccrl2*, *Ccl4*, and *Cxcl2*. Finally, the calvaria exhibited DE pro- and anti-inflammatory genes mostly in neutrophils, such as *Il1b*,^31^ *Ptgs2*,^32^ and *Thbs2*,^33^ of which some are also involved in cell adhesion and migration. In sham-condition calvaria, some genes were common with naive differentially upregulated genes (DUGs) such as *Nr4a1* and *Egr1* in addition to some distinct ones such as *Btg2* (anti-proliferation factor).^34^

In MCAo-condition calvaria, neutrophils harbored most DUGs. Some DUGs were in common with other conditions such as *Nr4a1*, *Cxcl2*, *Ccrl2*, and *Egr1*, whereas others were unique to stroke, such as *Cd69* (T cell migration),^35^ *Gpr35* (inflammation regulation),^36^ and *Nr4a3* (T cell and progenitor proliferation)^29^ (Figure 2C). We validated the upregulation of *Nr4a1* in the calvaria using tissue clearing and immunostaining (Figures 2D and 2E) and RNAscope^37^ (Figure 2F).

Focusing on the damage-associated molecular patterns (DAMPs) in CD45+ immune cells, known to guide the immune response in trauma and infection,^38^ we found calvaria-specific expressions of *Trem1*, *Trpm2*, *Nlrp3*, *Trem2*, and *Cgas* (Figure S3D). The skull was unique in downregulating *Trem1* and *Tlr2* in response to MCAo (Figure S3E).

On investigating the LR interactions using CellPhoneDB,^39^ we identified bone-type unique interactions (Figure 2G; Table S1, tabs 14–28). In each of the three conditions, we found a core module of LR interactions common to all bones. The numbers of common LR pairs increased for MCAo (naive, 1,153; sham, 1,163; and MCAo, 1,382 pairs). Among the unique interactions, calvaria had the most for naive and MCAo (153 and 73, respectively), whereas vertebra had the most in sham (173).

Gene ontology (GO)-term analysis showed that common pairs to all bones in all conditions were mostly involved in cell migration, cytokine production, and immune regulation such as *Pecam1-Cd177*,^40^ *Cd74-Mif*,^41^ and *Lgals9-Cd47* (Table S1, tabs 18, 23, and 28). The calvaria-unique pairs included *Il1b-Adrb2* and *Ccl4-Ccr5* in naive, *Ccl4-Cnr2* in sham, and *Cxcl2-Dpp4* and *Cd28-Cd86* in MCAo conditions. Naive LR pairs had GO terms mostly related to taxis, cell motility, and cytokine production whereas sham had immune activity-related terms (Figure S3F; Table S1, tabs 18, 23, and 28). Skull-unique LR pairs in MCAo were mostly related to cell migration, chemotaxis, or immune cell activation (Figure S3F).

In conclusion, calvaria displayed the highest number of DUGs and LR pairs among the bones tested, suggesting a distinct molecular profile related to migration and inflammation, especially in the myeloid lineage. This unique signature might underly the differential cell recruitment from the skull bone marrow to the brain.^2^^,^^4^^,^^5^^,^^6^

### Skull and meningeal neutrophils share unique similarities

As most of the calvaria-specific genes were in neutrophils (Figure 2B), we next examined their developmental trajectories using RNA velocity^42^ in its scVelo^43^ implementation and pseudo-time,^44^ which aligned well with the RNA velocity trajectory (Figures 2H and S3G). Our analysis revealed a subset of mature neutrophils from calvaria clustering next to a group of neutrophils found in the dura (Figure 2H). Along the trajectory, we observed a higher percentage of late neutrophils in the calvaria compared with other bones (Figure S3G). Upon injury, we observed a shift toward late neutrophils in the dura, most prominently in MCAo (Figure S2A). A representative phase portrait of a calcium-binding gene *S100a6* confirmed the validity of our scVelo trajectory analysis (Figure S3H).

To investigate the similarity of mature neutrophils in the calvaria and dura, we performed branching trajectory analysis using partition-based graph abstraction (PAGA).^45^ We observed a clear distinction between the naive vs. injury groups with the dura positioned in the middle (Figure 2I). The meningeal neutrophils from the naive condition connected with almost all bones in the naive condition, whereas the sham and MCAo meningeal neutrophils connected to the calvaria’s sham and MCAo, revealing a similarity between their late-stage neutrophil population profiles. The number of common DEGs between the dura and the calvaria also increased from 6 upregulated and 7 downregulated genes in naive (Figures 2J and S3I–S3K) to 29 upregulated to 15 downregulated genes in MCAo (Figure S3K).

The calvaria displayed the highest pro-inflammatory signature among bones in all conditions (Figure S3L) with neutrophils having the highest pro-inflammatory signature in the calvaria and B cells having the lowest (Figures S3L and S3M). Comparing the pro- and anti-inflammatory scores of the meningeal immune cells with those of the bones, we saw a stronger inflammatory response to injury and especially to MCAo in the meningeal cells, mainly in monocytes and neutrophils.

**Figure S4:**
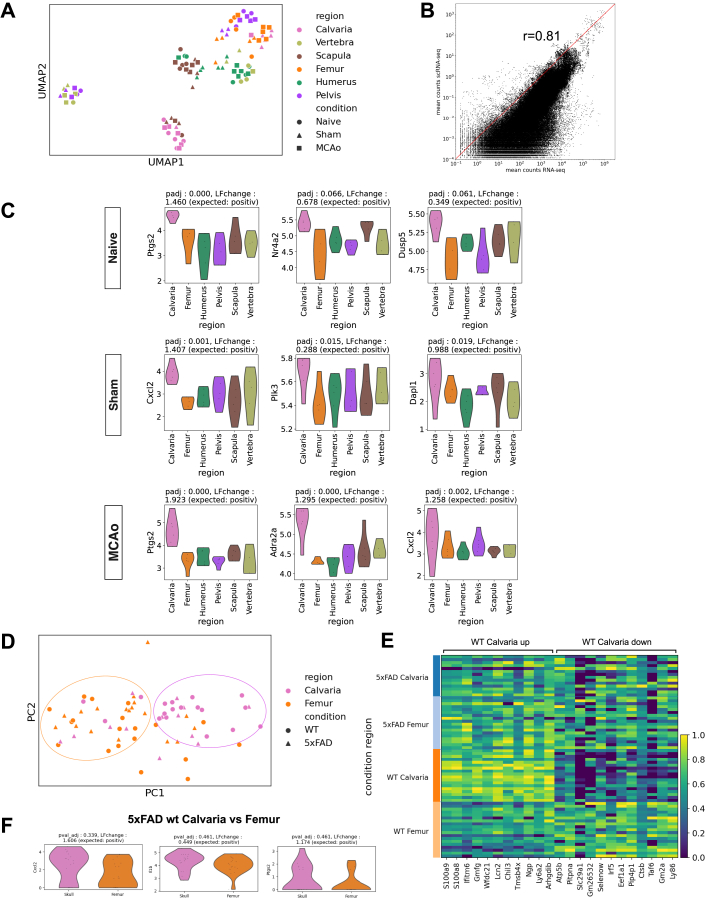
Analysis of bulk RNA-seq data of bone marrow cells, related to Figure 2 (A) PCA of calvaria, scapula, humerus, vertebra, pelvis, and femur from 5 naive, 5 sham, 6 MCAo animals. Color represents region and shape represents condition. (B) Correlation between bulk RNA gene expression and scRNA-seq pseudobulked dataset. r = 0.81. (C) Representative genes that show the same trend with scRNA-seq data for each condition. p values and log-fold changes are given on top of each violin plot (p < 0.001 for Ptgs2, p = 0.066 for Nr4a2, and p = 0.061 for Dusp5 in naive, p = 0.001 for Cxcl2, p = 0.015 for Plk3 and p = 0.019 for Dapl1 in sham, p < 0.001 for Ptgs2, p < 0.001 for Adra2a, and p = 0.002 for Cxcl2 in MCAo). Single-cell expression of these genes are given with “expected,” positive means scRNA-seq data showed an increased trend of the given gene. (D) PCA of femur and calvaria in 5xFAD model of Alzheimer’s disease. 5xFAD animals are compared with their littermate controls. Colors represent different bones whereas shapes represent condition. (E) Calvaria upregulated and downregulated genes in control case. There are no differentially expressed genes in AD case. The expression of the differentially expressed genes are shown in all groups for comparison. (p < 0.05) (F) Selected upregulated genes that show the same trend in 5xFAD dataset. p values and log-fold change are given on top of each violin plot (p < 0.339 for Cxcl2, p = 0.461 for Il1b, and p = 0.461 for Ptgs2 . Single-cell expression of these genes are given with expected, positive means scRNA-seq data showed an increased trend of the given gene.

We validated our scRNA-seq results using bulk RNA-seq for the same bones. Uniform manifold approximation and projection (UMAP) showed similar trends as we saw in scRNA-seq data (Figure S4A). The overall mean correlation of gene expression values between the bulk dataset and a pseudo-bulk created from the scRNA-seq dataset was r = 0.81 (Figure S4B). 69 of the 98 genes in naive, 19 of the 78 genes in sham, and 48 of the 62 genes significantly upregulated in calvaria in the pseudo-bulk scRNA-seq data showed the same trend in both datasets (Figure S4C). 9, 4, and 21 of these genes showed the same trend and were also significant in both samples for naive, sham, and MCAo conditions, respectively (Figure S4C; Table S1, tabs 31–34).

We also sequenced CD45+ cells in 6-month-old 5xFAD AD model mice vs. littermates using smart-Seq2 (n = 3 per group) (Figures S4D and S4E). Comparing smart-Seq2 data from wild-type calvaria and femur with our scRNA-seq dataset, we found that 15 of the 23 upregulated genes showed the same trend in both (Figure S4F; Table S1, tab 35).

Overall, our data show that bones change their transcriptome in pathologies, and the calvaria holds a distinct profile mostly close to meninges.

### Protein-level bone heterogeneity in mice

**Figure 3:**
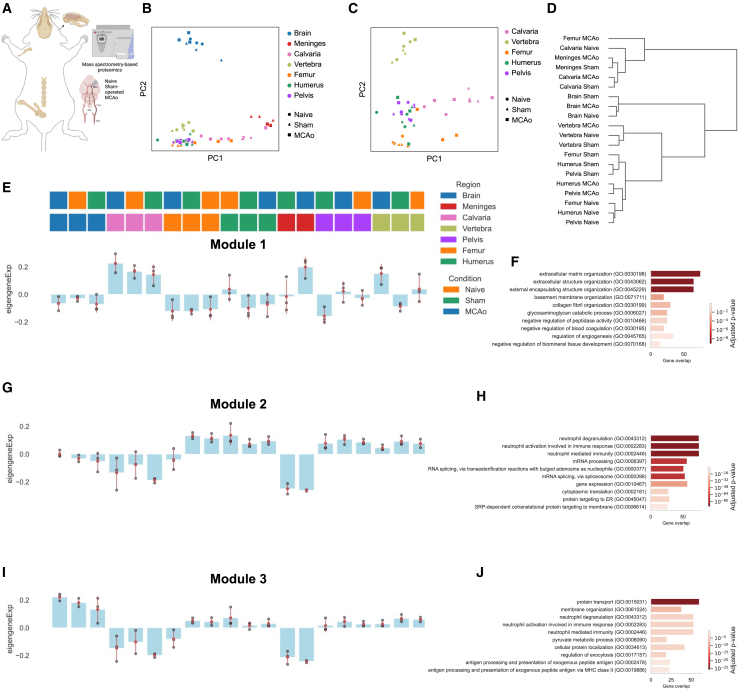
Proteomics identifies protein modules that characterize inter-bone expression differences (A) Illustration of the experimental pipeline is shown: mouse calvaria, humerus, vertebra, pelvis, and femur from three animals were collected to perform mass spectrometry in three different conditions, that is, naive, sham-operated, and MCAo. (B and C) Principal component analysis (PCA) of (B) six bones, dura, and brain and (C) six bones in naive, sham, and MCAo conditions. (D) Dendrogram demonstrates the relation among bones and conditions. (E–J) Protein expression modules identified by WGCNA among bones, brain, and meninges. Module distributions are shown in the left-hand panels the corresponding GO terms in the right-hand panels (n = 3 independent samples each for bones and brain for all conditions, n = 3 for meninges MCAo and sham conditions). See also Figure S5.

**Figure S5:**
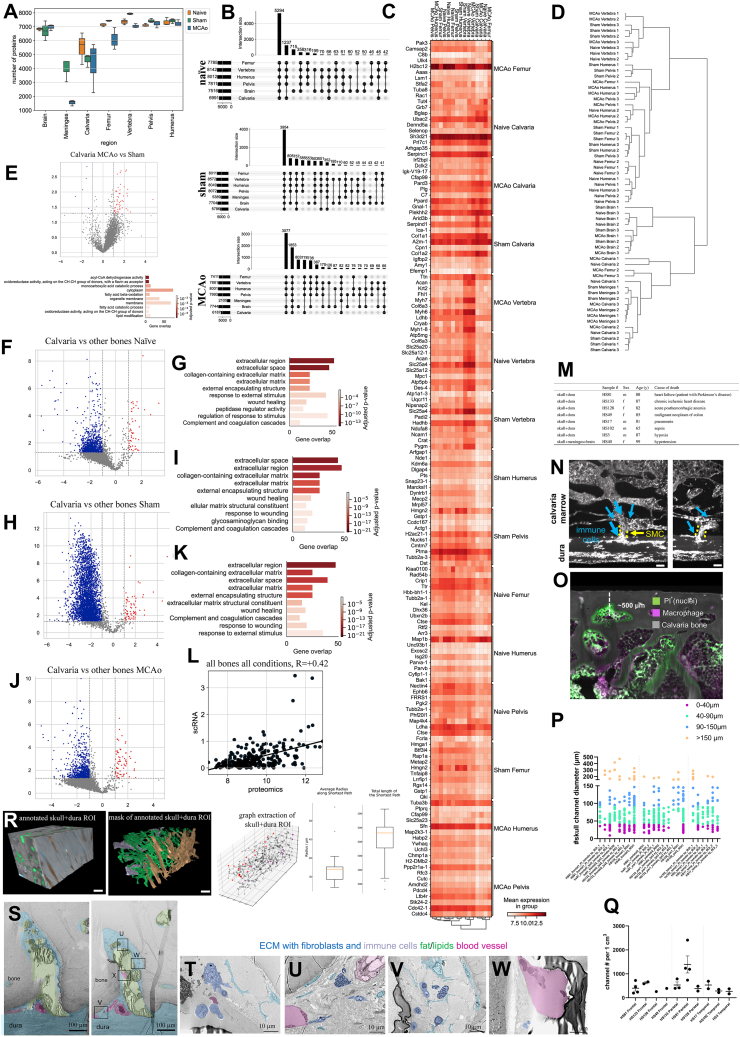
Details of the analysis of mouse proteome data and human skull-meninges channels, related to Figures 3 and 4 (A) Number of proteins detected from each bone. (B) Number of common proteins and unique proteins detected from different bones for different conditions. Top: naive, middle: sham, and bottom: MCAo. (C) 10 top upregulated proteins for each region in each condition (LFC > 1, p < 0.05). (D) Dendrogram for each sample and condition is shown. (E) Volcano plot shows the difference between calvaria MCAo vs. sham. (LFC > 1, p < 0.05) Related GO terms are shown below. (F–K) Volcano plots are showing (F) naive calvaria vs. other bones, (H) sham calvaria vs. other bones and (J) MCAo calvaria vs. other bones, respectively. (LFC > 1, p < 0.05) (G–K) GO terms of upregulated calvaria proteins in (G) naive, (I) sham, and (K) MCAo conditions are provided below each volcano plot. (L) Correlation plot of module 2 of WGCNA neutrophil degranulation GO term proteins with scRNA-seq expression levels. Spearman correlation, R = +0.42, p < 0.0001. (M) Details for post-mortem tissue clearing and light-sheet fluorescent imaging experiments. (N) Channels connecting calvaria’s bone marrow to the meninges with Iba1+ cells. Scale bars, 150 μm. (O) Human bone marrow labeled for cell nuclei (PI, in green), macrophage (Iba1, in magenta) is shown with calvaria bone (autofluorescence). (P) Skull channel diameter distribution based on each ROI quantified. (Q) Channel number per 1 cm^3^ distribution over all ROIs and samples. (R) Annotated skull + dura ROI, bottom part shows dura with brown annotation, skull channels are annotated in green and bone marrow is annotated in gray. Annotated dura, skull and bone marrow mask. Graph extraction of human skull architecture, total length, and radius of the shortest path from skull marrow to the dural meninges in μm, respectively. Scale bars, 500 μm. (S–W) 200 nm thick scanning electron microscopy images of a SMC with zoom-ins. (S) shows different axial depths of the same channel.

After transcriptomics, we also investigated proteome profiles in mouse bones, meninges, and brain using mass spectrometry proteomics (three biological replicates) (Figures 3A and S5). We quantified 9,597 proteins in total, 4,172 present in at least half of the samples (Figure S5A) and at least 5,000 proteins were present in at least one sample of each bone (Figures S5B–S5D).

Principal component analysis (PCA) showed segregation of the brain and meninges from bones. Calvaria samples were distributed over the PC1, clustering closest to the meninges (Figure 3B) and closer to femur in MCAo conditions (Figure 3C). We did not observe any clustering based on conditions (Figures 3B and 3C). On comparing the calvaria’s proteomic signature in sham and MCAo, we found 28 upregulated and 6 downregulated proteins (Figure S5E; Table S2, tab 10). Prominent examples include complement proteins such as CFB, which regulates B cell differentiation^46^ and cell adhesion factors including CD9^47^ and NID2^48^ (Table S2, tab 10).

A matrix plot and a dendrogram confirmed the segregation of calvaria, meninges, and femur MCAo samples from all other bones across all conditions (Figures 3D, S5C, and S5D). We found 45 upregulated proteins in the calvaria in naive condition (p < 0.005, log fold change [LFC] > 1), 65 proteins in sham, and 67 proteins in MCAo compared with other bones, whereas we identified a higher number of downregulated proteins (Figures S5F–S5K; Table S2, tabs 1, 4, and 7).

Using weighted correlation network analysis (WGCNA), we identified three modules with calvaria-specific differences. Module 1 was (mostly related to extracellular matrix [ECM] organization) increased in calvaria samples for all three conditions as well as meninges MCAo and vertebra MCAo samples (Figures 3E and 3F). Module 2 (mainly involved in neutrophil degranulation and immunity, and mRNA processing) showed a decreased expression in brain, calvaria, and meninges (Figures 3G and 3H). Module 3 was also downregulated in calvaria and meninges (Figure 3I) with GO terms related to protein transport, neutrophil degranulation, and immune pathways (Figure 3J). Comparing the protein with scRNA-seq data for module 2, we found a Spearman correlation value of R = 0.42, suggesting that this phenomenon is recapitulated on the RNA level^49^ (Figure S5L). Our proteomic analysis confirms neutrophils as a major source of the differences between calvaria and the other bones.

### Characterization of SMCs in human samples

**Figure 4:**
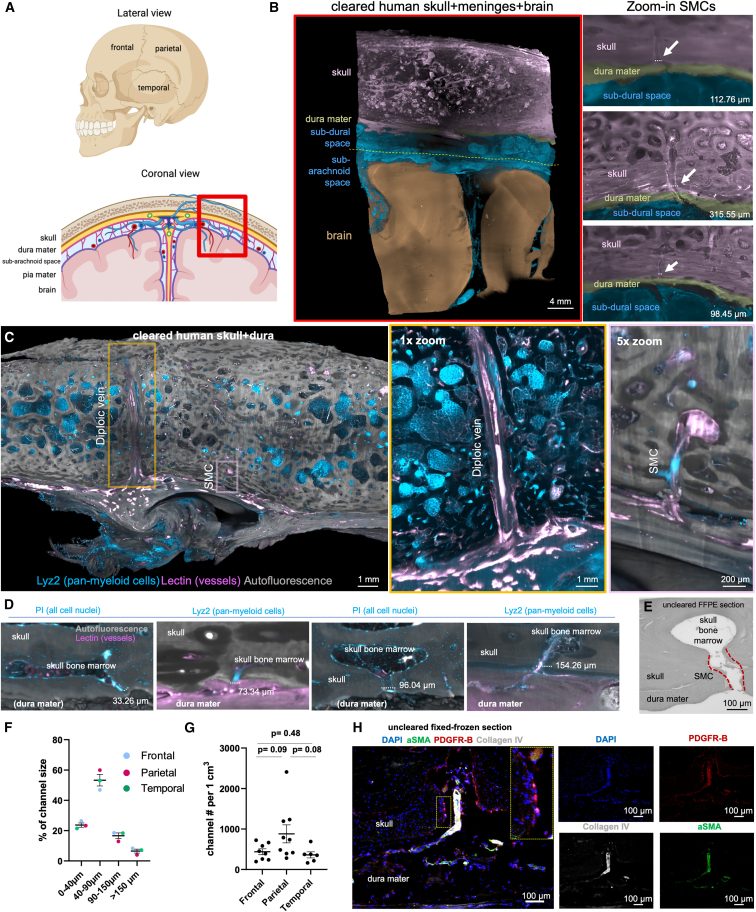
Tissue clearing enables a comprehensive characterization of human skull-meninges connections (A) Frontal, parietal, and temporal regions of the skull and coronal view depicting the meningeal layers and the brain. (B) Representative light-sheet microscopy image of cleared tissue corresponding to the red box in (A). The right panels show skull-meninges channels connecting the skull bone marrow to the sub-dural space and to the dura mater. (C) Representative skull piece cleared and imaged for SMC quantification in different regions of the human skull. Diploic vein and an exemplary SMC are shown. (D) Representative skull-meninges-channels in different sizes: ∼33, ∼73, ∼96, and ∼154 μm. Autofluorescence in gray, lectin in magenta. Left panels are labeled with PI (cyan) and right panels with LYZ2 (cyan). Dura mater in some panels is not preserved in (D). (E) Human SMC example from 1 μm thick FFPE embedded skull-dura section. (F) Quantification for % of channel size in frontal, parietal, and temporal regions. Data represented as ± SEM. (G) Quantification for annotated channel numbers, normalized to 1 cm^3^ (22 region of interests (ROIs) in total, >500 channels, from seven post-mortem samples, frontal vs. parietal p = 0.09, parietal vs. temporal p = 0.08, and frontal vs. temporal p = 0.48). Data represented as ±SEM. (H) Human SMC example with an artery passing to the skull from 8 μm thick fixed-frozen skull-dura section labeled with DAPI (blue), aSMA (green), PDGFR-B (red), and CollagenIV (gray). See also Figure S5.

We next explored the relevance of our findings in humans. First, we characterized SMCs using tissue clearing and light-sheet fluorescent imaging in 23 skull + dura mater samples in frontal, parietal, and temporal regions coming from seven human skulls (Figures 4A and S5M). We used immunofluorescence to label myeloid cells (LYZ2) and macrophages (IBA1) (Figures S5N and S5O), PI to label cell nuclei, and lectin to label vasculature (Figures 4B–4D). Human SMCs most often transverse the dura mater, opening to the sub-dural space underneath to arachnoid granulations^50^ (Figure 4B; Videos S2 and S3). We confirmed that SMCs transverse the dura using bright-field imaging of uncleared formalin-fixed paraffin-embedded (FFPE) sections of decalcified human skull (Figure 4E).

Video S2. Part (1) Tissue cleared and light sheet imaged human skull-meninges-brain block demonstrates the channel structure; part (2) IBA1+ labeled human skull+meninges (dura) sample displays immune cells in the skull-meninges connections; part (3) LYZ2 and lectin labeling on the human skull + dura sample shows an overview of skull structure, demonstrates channels going through the dura, related to Figure 4

Video S3. LYZ2 and lectin labeling on human skull + dura sample shows an overview of skull structure and demonstrates a diploic vein and SMCs, related to Figure 4

We quantified more than 500 SMCs and found that they are mostly 40–90 μm wide (Figures 4F and S5P) as suggested.^4^ Some SMCs were >150 μm, which were often surrounding big blood vessels and occasionally diploic veins (Figure 4C; Video S3). Region-based analysis did not reveal significant differences (Figures 4G and S5Q). We next used graph analysis and found the average shortest path length from a bone marrow cavity to SMC as ∼3,000 μm, and the average radius along the shortest path as ∼37 μm (Figure S5R). Furthermore, using histology on skull + dura mater, we found that PDGFR-B signal was present both at the vessels and at the SMC lumen (Figure 4H). This suggests that the SMC lumen is lined with a layer of fibroblastic cells, known antigen-presenting cells,^51^ that might potentiate cerebrospinal fluid (CSF) sampling already at the beginning of the SMCs.

Finally, we performed scanning electron microscopy on human skull + dura mater (Figures S5S–S5W). We found similar structures as we identified using tissue clearing, immunohistochemistry (IHC), and as previously shown using microCT^4^ that were filled with fat/lipids. The SMC structure showed immune cells within, in addition to a fibroblastic cell layer (Figures S5S–S5W) as suggested by histology (Figure 4H). These findings suggest that human SMCs might be filled with fat, unlike those of mice,^4^ allowing immune trafficking while serving as an energy source to hematopoietic stem cells.^52^^,^^53^^,^^54^

### Human skull proteome is distinct from vertebra and pelvis

**Figure 5:**
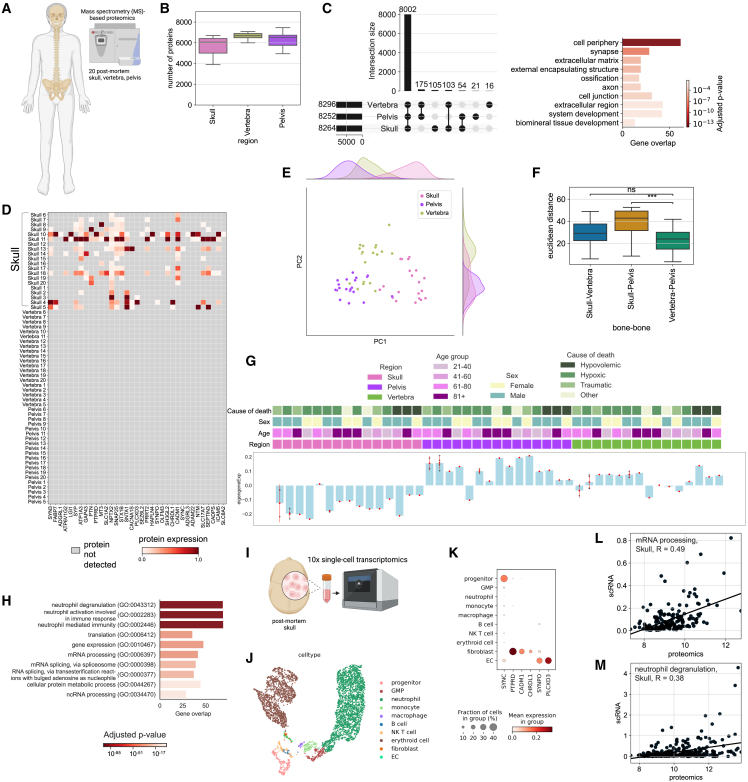
Human bones differentially express distinct protein modules (A) Illustration of the experimental pipeline, 60 bones in total were collected to perform mass spectrometry-based proteomics on 20 skull, 20 vertebra, and 20 pelvis. (B) The number of proteins detected from each bone is shown with a boxplot. (C) The number of common proteins and unique proteins detected from different bones are shown with an upset plot. GO terms associated with unique skull proteins are shown at the bottom. (D) Expression levels of a selection of proteins belonging to GO terms related to synapse term that were detected in more than half of the skull samples uniquely. (E) Principal component analysis of the three bones analyzed. (F) Boxplot depicts the Euclidean distances between pairs of bones using the first 2 principal components. (p = 2.862e-04 for skull-pelvis vs. vertebra-pelvis, p value =2.862e-04 for skull-pelvis vs. vertebra-pelvis) (G) WGCNA among bones reveal one significant module where calvaria genes are downregulated compared with two other bones with some exceptions. Biggest source of variance is the bone type. (H) GO terms from the module of skull downregulated proteins. (I) Single-cell sequencing of post-mortem skull sample illustration. (J) UMAP of single-cell sequencing of post-mortem skull sample (n = 1). (K) Expression of unique skull detected proteins in the scRNA-seq data. (L) Correlation plot of the module from (G), mRNA processing GO term. Protein expression vs. scRNA-seq. Spearman correlation, R = 0.49, p < 0.0001. (M) Correlation plot of the module from (G), neutrophil degranulation GO term protein expression. Protein expression vs. scRNA-seq. Spearman correlation, R = 0.38, p < 0.0001. See also Figure S6.

**Figure S6:**
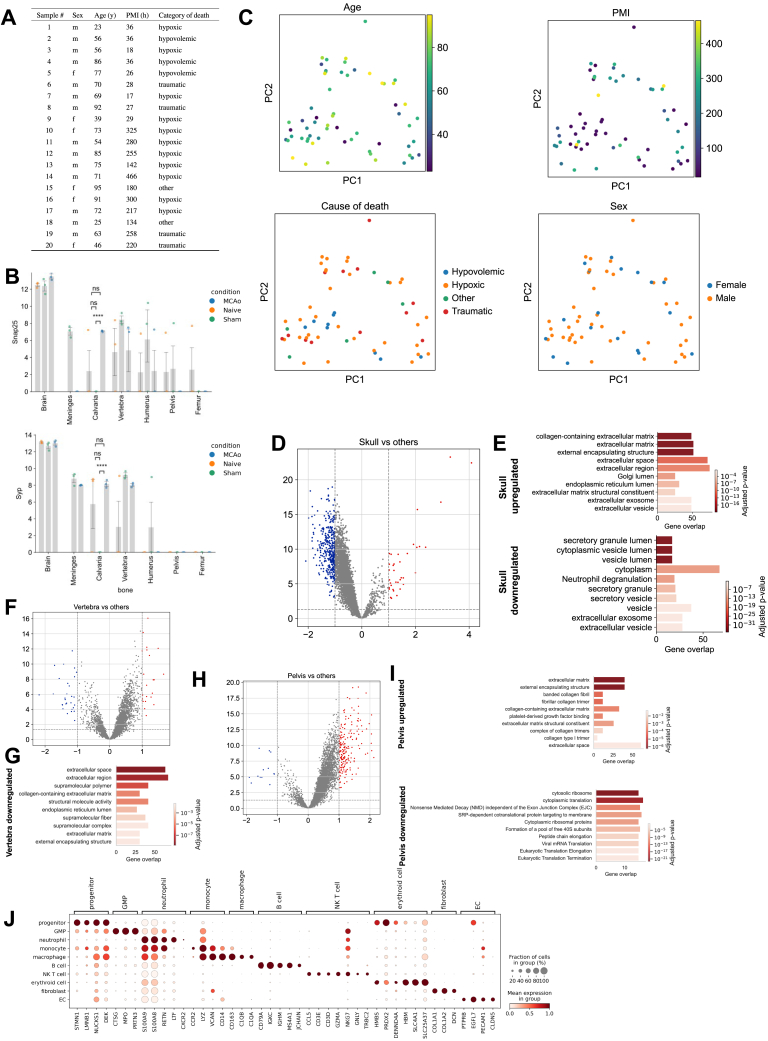
Details of the analysis of the human proteome data, related to Figure 5 (A) Post-mortem sample information, category of death is based on how death affects the brain. (B) Two proteins found uniquely in the human skull that show a similar trend in the mouse dataset. Snap25 and Syp expression in calvaria MCAo is higher than in sham (p = 5.786e-08 and p = 2.000e-05, respectively). (C) PCA of bones based on age, cause of death group, PMI, and sex, respectively; PMI, post-mortem interval. (D–I) Volcano plots among different bones: calvaria vs. others (D), vertebra vs. others (F), and pelvis vs. others (H) suggest there is a global downregulation in the skull compared to pelvis. (LFC > 1, p < 0.05) with GO terms for upregulated and downregulated for each bone (E), (G), and (I). (J) Cell-type annotation marker genes for scRNA-seq of human skull.

Next, we obtained 20 post-mortem human skull, vertebra, and pelvis samples from two independent autopsy centers for proteomic analysis (Figures 5A and S6A). We detected 8,526 protein groups before and 5,320 protein groups after filtering (Figure 5B).

The highest number of uniquely detected proteins was in the skull with 105 unique proteins (Figure 5C). GO analysis revealed 27 skull-specific synapse and synaptic signaling related terms. For example, the term “chemical synaptic transmission” was represented by proteins such as SYP, SYN3, SNAP25, and SLC17A7 (Figure 5D; Table S2, tabs 14–16). Mouse proteome also showed a positive trend in *Syp* and *Snap25* proteins in the calvaria (Figure S6B). This might suggest that skull is more involved in neuropeptide or neurotransmitter-based communication, compared with other bones.^55^

The PC1 of PCA plot depicts the distinct nature of the skull, whereas PC2 demonstrates that the skull samples have a larger variance (Figure 5E). Euclidian distance between pairs showed the differences between skull and pelvis to be the highest and that between vertebra and pelvis to be the lowest (Figure 5F). Bone proteome differences were not driven by age, post-mortem interval, sex, or cause of death (Figure S6C). On the global proteomic scale, we observed a strong downregulation of proteins in the skull compared with other bones, few DE proteins between the vertebra and the other bones, and a strong upregulation of proteins in the pelvis (Figures S6D–S6I; Table S2, tabs 19–27).

Cellular processes such as translation, metabolism of RNA, and leukocyte activation-related terms were downregulated in the calvaria, whereas ECM organization-related terms were upregulated (Figure S6E; Table S2, tab 20). These differences are in line with our mouse scRNA-seq dataset, e.g., in the collagens *Col1a1* and *Col1a2* in naive (p = 0.0004 and p = 0.0016, respectively) and MCAo (p = 0.00005 and p = 0.0002, respectively) conditions. In mice, *COL1A1* and *COL1A2* were also among the top DE proteins in the calvaria (Table S2, tabs 1, 4, and 7). ECM strongly influences immune cell functions,^56^ suggesting that the functional role of ECM differences should be investigated further. Additionally, the most abundant protein in our human skull dataset was *COL1A1*, a structural protein encapsulating blood vessels in bone marrow,^57^ suggesting differences in the vascular organization of the skull bone marrow.

Using WGCNA, we identified a module that was downregulated in the skull samples of the human proteomics dataset (Figure 5G), whose GO terms were very similar to mouse proteome modules: most prominently neutrophil degranulation and mRNA processing (Figure 5H).

Overall, two notable groups of proteins showed interesting expression profiles between the bones. First, we identified several proteins unique to the skull that relate to synapses, and second, a downregulation of neutrophil degranulation and mRNA processing in the skull. To test how these differences would translate into the RNA level, we performed scRNA-seq of one human post-mortem skull (Figure 5I). After annotating 10 cell types (Figures 5J and S6J), we assessed the presence and expression levels of unique skull proteins (Figure 5C). Six of the 256 unique synapse-relevant genes were detected in the dataset, mostly in fibroblasts (Figure 5K). This allowed us to rule out immune cell expression as a source of the synapse-related terms. We speculate that the difference in synaptic protein levels could either hint at a denser or more active innervation of the skull bone marrow or it might reflect the immune surveillance of the brain that leads to a transport of peptides from brain to the skull.

The human scRNA-seq data supported the presence of the mRNA processing and neutrophil degranulation modules. Proteomics data and scRNA-seq data correlated with R values of R = 0.49 and R = 0.38 for the genes in these GO terms, respectively (Figures 5L and 5M). This correlation from both mouse and human datasets suggests a consistently low neutrophil degranulation and lower mRNA processing, based on previously reported correlations between mRNA-protein levels.^58^^,^^59^ Thus, we conclude that the human skull differs from other bones at both transcriptomic and proteomic levels.

### TSPO signal in the skull is associated with inflammatory, ischemic, and neurodegenerative CNS diseases

**Figure S7:**
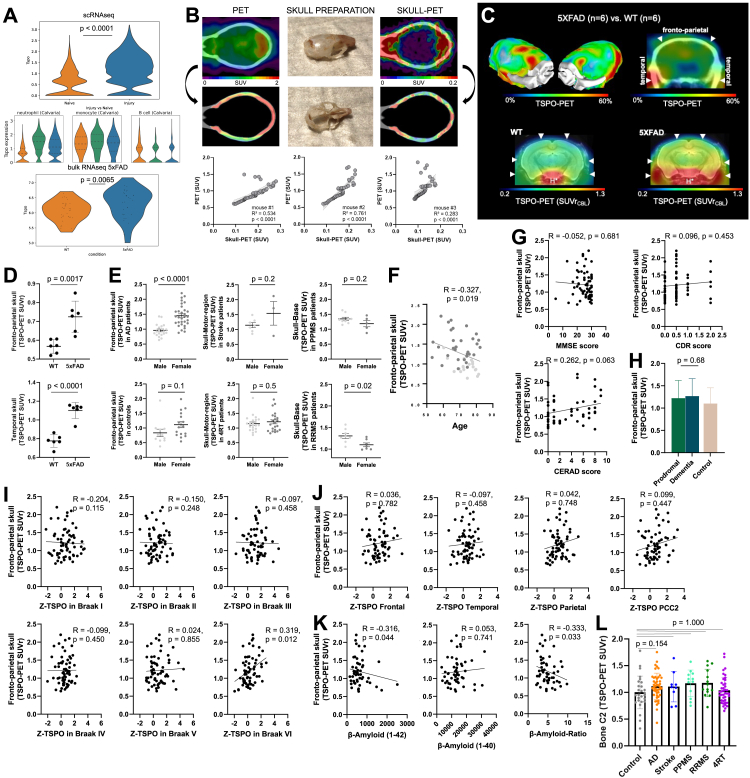
Influence of imaging method, and various covariates on TSPO-PET data, related to Figure 6 (A) TSPO RNA levels in naive vs. injury (MCAo + sham) (p < 0.0001) conditions in the skull from the scRNA-seq data. TSPO RNA levels in 5xFAD vs. wild type in the calvaria (p = 0.0065). (B) *In vivo* TSPO-PET imaging of three wild-type mice, followed by a second scan after immediate removal of the brain, blood, and all tissue surrounding the skull bone. Signal attributable to the skull in the *in vivo* TSPO-PET images was compared to the signal in the respective skull-only TSPO-PET to delineate skull signal in mice (three replicates, R² = 0.534, 0.761, 0.283, p < 0.0001). (C) Coronal slice upon a CT template shows %-TSPO-PET differences between 5xFAD and wild-type mice at the group level. Images indicate increased TSPO labeling in the fronto-parietal and temporal skull of 5xFAD mice in contrast against age-matched wild-type mice. White arrows indicate spots with higher increases of skull TSPO labeling when compared to adjacent increases of brain TSPO labeling in 5xFAD. Axial slices upon an MRI template show TSPO-PET in an individual 5xFAD and an individual wild-type mouse. Elevated TSPO labeling in fronto-parietal and temporal skull is present (white arrows) in the 5xFAD mouse when compared to the wild-type mouse. H^∗^ = hypophysis with known strong TSPO-PET signal. (D) Fronto-patietal skull, p = 0.0017, temporal skull, p < 0.0001 (two-tailed t test). Data represented as ±SEM. (E) Quantification of relevant skull signal sex differences for AD (p < 0.0001; controlled for age and TSPO-binding single nucleotide polymorphism), stroke (p = 0.2), PPMS (p = 0.2), RRMS (p = 0.02), 4RT (p = 0.5) patients and controls (p = 0.1). Data represented as ±SEM. (F) Quantification of fronto-parietal skull signal age associated patterns (p = 0.019, two-tailed t test, controlled for gender and TSPO-binding single nucleotide polymorphism) among 50 AD continuum patients. Data are means ± SD. SUVr, standardized uptake value ratio. (G) Fronto-parietal skull TSPO signal from patients with AD show no significant correlation with clinical severity in MMSE (p = 0.681), CERAD (p = 0.063), and CDR (p = 0.453) scorings. (H) Fronto-parietal skull TSPO signal in Alzheimer’s disease compared to control patients (prodromal vs. dementia: p = 0.63, data represented as ±SEM.). (I) Fronto-parietal skull TSPO signal shows a positive association only with brain TSPO signal in the Braak VI stage region (p = 0.115 for Braak I, p = 0.248 for Braak II, p = 0.458 for Braak III, p = 0.450 for Braak IV, p = 0.855 for Braak V, and p = 0.012 for Braak VI). (J) Fronto-parietal skull TSPO signal is not significantly associated with brain TSPO signal in any β-amyloid related regions: frontal (p = 0.782), temporal (p = 0.458), parietal (p = 0.748), and posterior cingulate cortex/precuneus (p = 0.447). (K) Fronto-parietal skull TSPO signal is correlated with β-amyloid_42_ (p = 0.044) but not β-amyloid_40_ (p = 0.741) in cerebrospinal fluid, also reflected by the significant negative correlation of the β-amyloid ratio (p = 0.033). (L) TSPO-PET signal quantifications in C2 bone of vertebra. One-way ANOVA with Bonferroni post hoc correction. See STAR Methods for details of normalization and statistical analysis. Significant differences of disease vs. controls are indicated (p = 1.0 for control vs. stroke, PPMS, RRMS, and 4RT, p = 0.154 for control vs. AD). Data represented as ±SEM. Pairwise comparison of all groups can be found in Table S3.

Next, we examined the reaction of the skull to different neurological disorders in patients. TSPO is a protein markedly upregulated in the brain during neuroinflammation and is used as a PET biomarker.^60^^,^^61^ We also found significantly higher *Tspo* RNA levels in the calvaria in injury compared with naive mice (Figure S7A), especially in neutrophils. To confirm the ability of PET imaging to measure skull-specific TSPO-PET signals, we performed imaging on three living mice and immediately isolated the skulls. The isolated skulls had a strong association with the skull signal in the live animals confirming the skull origin of the TSPO signal (Figure S7B).

**Figure 6:**
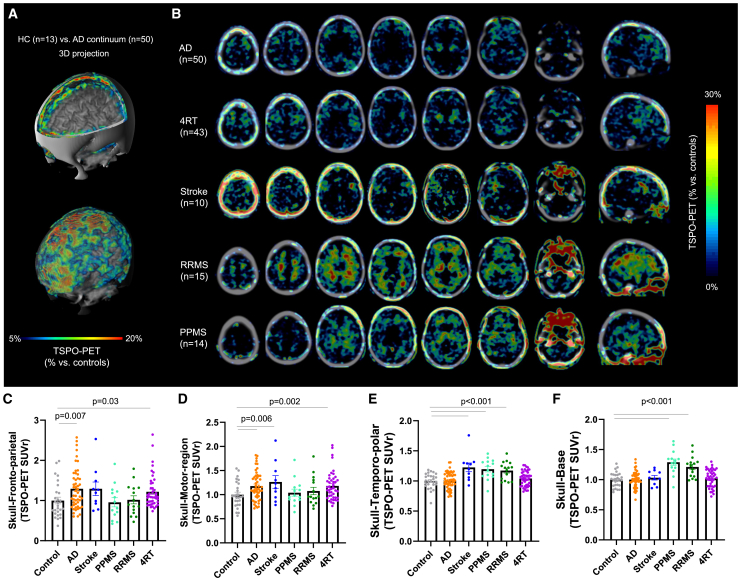
Distinct TSPO uptake patterns are observed in the skull of patients with inflammatory, ischemic, and degenerative CNS diseases (A) 3D surface projection (triple fusion with CT and MRI templates; quadrant cut [top]; transparent CT [bottom] displaying increased activity within skull) shows %-TSPO-PET differences between patients with AD and healthy controls at the group level. (B) Average TSPO-PET signal in Alzheimer’s disease (AD), stroke, primary progressive multiple sclerosis (PPMS), relapsing-remitting multiple sclerosis (RRMS), and 4-repeat tauopathy (4RT) patients. (C–F) TSPO-PET signal quantifications in skull regions adjacent to different brain regions: (C) fronto-parietal area (p = 0.007 for control vs. AD, and p = 0.03 for control vs. 4RT), (D) motor area (p = 0.006 for control vs. AD and stroke, and p = 0.002 for control vs. 4RT), (E) temporopolar area (p < 0.001 for control vs. stroke, PPMS, and RRMS), and (F) skull base (p < 0.001 for control vs. PPMS and RRMS). Data represented as ±SEM. One-way ANOVA with Bonferroni post hoc correction (see STAR Methods for details). Data were normalized as described in the STAR Methods. Significant differences of disease vs. controls are indicated. Pairwise comparisons of all groups can be found in Table S3. See also Figure S7.

Next, we assessed TSPO-PET signals in 50 patients belonging to the AD continuum, 43 patients with 4-repeat tauopathies (4RTs),^62^ 10 patients in the post-acute phase of stroke, 15 patients with relapsing-remitting multiple sclerosis (RRMS),^63^ and 14 patients with primary progressive multiple sclerosis (PPMS) (Table S3, tab 1). We used 3D surface projections on a CT template to show substantial relative TSPO-PET differences in patients belonging to the AD continuum compared with healthy controls (Figure 6A). We found a clear increase in TSPO-PET signals in calvaria regions adjacent to the frontal, parietal, and motor cortices of patients belonging to the AD continuum (Figures 6A–6F; Video S4). Similarly, elevated skull inflammation was observed in each cohort of patients with distinct patterns in different pathological conditions (Figures 6B–6F), e.g., a prominent temporal pole signal in stroke and multiple sclerosis patients (Figures 6B and 6E), in the skull base in RRMS and PPMS patients (Figure 6D), and in the skull adjacent to the prefrontal cortex and the motor area in 4RT patients (Figures 6C and 6D). In 5xFAD mouse model of AD, we observed a similar TSPO signal elevation in the fronto-parietal and temporal regions compared with controls (Figures S7C and S7D). These results indicate that TSPO-PET imaging of the skull can reveal distinct signal patterns in inflammatory, ischemic, and degenerative CNS conditions, at least at the cohort level.

Video S4. TSPO-PET signal from an Alzheimer’s patient shows a high TSPO-PET signal coming from the calvaria, related to Figure 6

In the AD continuum patients, the overall TSPO-PET signal was increased in females over males and was negatively associated with age in patients with AD (Figures S7E and S7F). We did not find statistically significant differences between male and female patients in the 4RT, stroke, and PPMS cohorts, although RRMS showed increased TSPO tracer uptake in males (Figure S7E). We did not find significant correlations with AD severity based on cognitive tests such as mini-mental-state examination (MMSE), the Consortium to Establish a Registry for AD (CERAD) neuropsychological test battery, and the clinical dementia rating (CDR) scale (Figure S7G). There were also no significant associations with specific clinical stages of AD such as in the comparison of the prodromal stage characterized by subjective cognitive decline (SCD) or mild cognitive impairment (MCI) and the AD dementia stage (Figure S7H). Early and late clinical AD subgroups displayed a similar increase in the calvaria TSPO-PET signal (Figures S7G and S7H), suggesting that skull inflammation occurs during all stages of the AD continuum.

Notably, a significant correlation between the TSPO-PET signals in the calvaria and the brain was only observed in Braak stage VI regions, which can suggest an increasing skull inflammation with advanced tau spread (Figure S7I). TSPO-PET levels in the calvaria were associated with decreased β-amyloid_42_ but not β-amyloid_40_ concentration in CSF (Figures S7J and S7K). Lower β-amyloid_42_ in CSF is associated with more fibrillar amyloid deposits in the brain,^64^ suggesting that β-amyloid is also a trigger for increasing skull inflammation. By comparison, the C2 bone of the vertebra had no significant increase compared with controls in any of our cohorts (Figure S7L).

**Figure 7:**
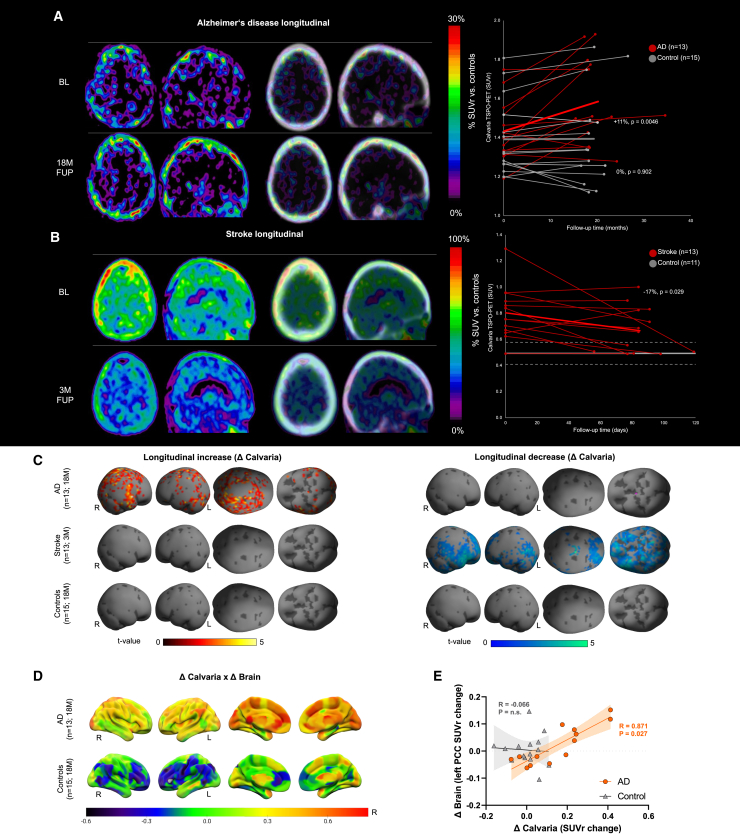
Serial calvaria TSPO-PET imaging of patients with Alzheimer’s disease and stroke (A and B) Axial and sagittal slices show %PET difference images of patients with Alzheimer’s disease (AD, n = 13, A, +11%, p = 0.0046 in AD vs. 0%, p = 0.902 in controls) and stroke (n = 13, B, -17%, p = 0.029 in stroke) against age-matched healthy controls (normalized as described in the STAR Methods). Controls in (A) (n = 15) were imaged serially and controls in (B) (n = 11) were imaged at a single time point. %PET difference images are depicted with and without CT overlay. Right panels show individual time courses of calvaria TSPO-PET signals of (A) patients with Alzheimer’s disease and healthy controls at a median follow-up interval of 18 months and (B) patients with stroke at a median follow-up interval of 84 days. Mean (thick line) and standard deviation (dashed lines) of calvaria TSPO-PET. (C) Surface projections show statistical parametric mapping (SPM) of longitudinal TSPO-PET changes (left: increases, hot/right: decreases, cold) of patients with AD, patients with stroke, and healthy controls. Voxels with p < 0.05 (t value threshold 1.78, uncorrected for multiple comparisons) are projected on the SPM12 skull surface template. (D) Brain surface projections show regional correlations (Pearson’s correlation coefficient, R) of longitudinal TSPO-PET changes in calvaria with longitudinal TSPO-PET changes in brain of patients with AD and healthy controls. (E) Correlation between calvaria and brain TSPO-PET changes in the left posterior cingulate cortex that survived false discovery rate correction for multiple comparison of 246 brain regions (R = 0.871, p = 0.027 in AD vs. R = -0.066, p = n.s. in controls).

Next, we performed longitudinal analysis on patients with stroke and AD. Our stroke patients were scanned again 3 months after the stroke, whereas patients with AD were imaged 18 months after their baseline scan. Time points were chosen based on clinical necessity. On comparing 13 patients with AD with 15 serially imaged age-matched healthy controls, we found, on average, an 11% increase in the skull TSPO-PET signal in patients with AD (p = 0.0046, paired t test), whereas healthy controls revealed no change (p = 0.902, paired t test, Figure 7A). By contrast, we observed a 17% decrease (p = 0.029, paired t test) in the skull TSPO-PET signal of stroke patients 3 months after the onset of their stroke (13 stroke patients, 11 controls) (Figure 7B).

After normalization (see STAR Methods), we generated surface projections to reflect the pattern of longitudinal TSPO-PET changes by statistical parametric mapping (SPM) (voxels with p < 0.05, uncorrected for multiple comparisons) (Figure 7C) on SPM12 skull surface template. We observed that signal increases in patients with AD were mainly observed in areas highly relevant to AD pathology such as the skull covering the temporal and parietal cortices^65^ and were also in line with the regions we report as significantly higher in the baseline condition, i.e., motor area and fronto-parietal cortex (Figures 6C and 6D). In stroke patients, we observed a longitudinal decrease in the whole skull without specific regional preferences (Figure 7C).

We correlated TSPO-PET changes in 246 brain regions of the Brainnetome Atlas^66^ with the overall skull TSPO-PET signal changes in patients with AD and found that regional increases of microglial activation in the brain are correlated with progressive overall skull inflammation (Figure 7D). This association was found for brain regions highly relevant to AD pathology, namely the posterior cingulate cortex (PCC), which remained statistically significant even after strict false discovery rate (FDR) correction for multiple comparisons (Figure 7E). Only weak correlations were found in controls (Figure 7D). These results suggest that skull responds to brain inflammation in AD and may serve as a proxy for monitoring neuroinflammation in humans.

## Discussion

Neuroinflammation is a significant factor in many CNS disorders. Recent findings suggest that studying the connections between the dura mater and neighboring calvaria marrow could provide insights into brain inflammation. Observing cells and molecules specifically associated with the calvaria-meninges-brain axis could be an effective way to monitor and understand this process.

Our study shows that there is a clear difference between the marrow cells suggesting localized functions for different bones. Our human proteomics analysis confirmed that human skull has a distinct molecular profile as in mice. As these proteomics data were obtained from post-mortem individuals with a wide range of ages and pathologies, it suggests that the skull bone marrow remains distinct across a wide range of conditions. Moreover, the increased levels of brain-related, especially synaptic proteins, in the human skull suggests that communication along the skull-meninges-brain axis might occur in both directions.^55^

Overall, our extensive data on human SMCs using tissue clearing of large samples of human skulls with attached dura mater suggest that they facilitate immune cell trafficking between the skull and meningeal surface of the brain similar to detailed observations provided in different studies in mice.^2^^,^^4^^,^^5^^,^^6^ Although the disruption of the blood-brain barrier after pathological conditions such as stroke as well as the infiltrating immune cells through blood has been well-documented,^12^^,^^67^^,^^68^ it is currently still unclear under which conditions and what fraction of immune cells reach the meninges or to the parenchyma from the calvaria compared with other routes.^6^^,^^12^^,^^69^

Several studies demonstrated the clinical utility of TSPO-PET imaging in neurological diseases such as AD, Huntington’s disease (HD), amyotrophic lateral sclerosis, Parkinson’s disease, multiple sclerosis, and migraine.^70^^,^^71^ The correlations between brain and skull inflammation signal we observed with a third-generation TSPO-PET ligand [^18^F]GE-180^72^ suggest that the use of skull imaging for the early diagnosis and/or monitoring of brain pathologies should be further investigated. However, given the limited disease specificity of the TSPO signal, it is likely that different contrast agents and imaging modalities will be needed for clinical applications. Our data support the notion of chronic inflammation in patients with AD^73^^,^^74^ vs. resolving inflammation in stroke patients after acute trauma.^75^^,^^76^ As calvaria cells are localized very close to the surface, it could be easier and faster to image it by different modalities, for example, optoacoustic imaging technologies in the future, which are portable and less costly compared with MRI/PET imaging and could provide early point-of-care diagnosis.^77^

TSPO is strongly upregulated in microglia and astrocytes upon activation and is also expressed on infiltrating macrophages in the brain.^60^ However, its sources outside the brain are less understood as many immune cell types including neutrophils express TSPO.^78^^,^^79^ The increased cell numbers in the skull marrow in response to injury also suggest a mechanism for the increase in TSPO signals seen in mouse and human data for various diseases, although more detailed studies are needed to establish the sources of signal increase for each condition.^80^^,^^81^

Our data suggest that different bones in the body have distinct molecular profiles. Notably, the response of the calvaria to neurological pathologies is different from other bones, indicating that the skull may be useful for monitoring and potentially controlling inflammation in various brain pathologies in the future.

### Limitations of the study

Our data have limitations, despite the rich data on molecular analysis and imaging in mice and humans. We could not statistically compare transcriptomics differences in mice and humans due to the limited number of samples. We only focused on a selected number of bones in mice and humans. Also, for ethical reasons, we could not obtain bone samples from healthy humans for comparison with pathological states. Different causes of death might also be affecting the molecular profile of the samples.

Although our data provide leads for the molecules that might regulate the skull’s response to disease, only future mechanistic studies can clarify their exact involvement. Future work must explore inter-individual differences and gene expression profile evolution over time in mice. Additional characterization of the specific contribution of skull immune cells compared with other bones and exact routes of trafficking is necessary to understand the neuroimmune axis. Although our study suggested B cell, T cell, and myeloid cell trafficking, it would be interesting to elucidate specific cell-type dynamics in certain disease cases. Finally, our detailed demonstration of skull inflammation in diverse diseases in humans suggests that it can be used for diagnosing or monitoring diseases in the future, but detailed clinical studies are needed to explore its clinical utility.

## STAR★Methods

### Key resources table

**Table:** 

REAGENT or RESOURCE	SOURCE	IDENTIFIER
**Antibodies**		

Atto647NconjugatedantiGFP nanobooster	Chromotek	Cat.#gba647n-100;RRID:AB_2629215
Anti-NUR77 antibody	Abcam	Cat# ab153914
anti-Lysozyme antibody	Abcam	Cat#ab108508; RRID:AB_10861277
Goat anti-Rabbit IgG (H+L) Highly Cross-Adsorbed Secondary Antibody, Alexa Fluor 647	Abcam	Cat#A-21245; RRID:AB_141775
APC/Cyanine7 anti-mouse Ly-6G/Ly-6C (Gr-1) Antibody	Biolegend	Cat#108423; RRID:AB_2137486
APC anti-mouse CD11c Antibody	Biolegend	Cat# 117309; RRID:AB_313778
BD Horizon™ BUV395 Rat Anti-Mouse CD45	BD Biosciences	Cat#565967; RRID:AB_2739420
BD Horizon™ BV421 Rat Anti-Mouse CD117	BD Biosciences	Cat# 562609; RRID:AB_11154585
BD Pharmingen™ PerCP-Cy5.5 Rat Anti-Mouse F4/80	BD Biosciences	Cat# 567202; RRID:AB_2916500
Brilliant Violet 510™ anti-mouse I-A/I-E Antibody	Biolegend	Cat# 107635; RRID:AB_2561397
Brilliant Violet 650™ anti-mouse TER-119/Erythroid Cells Antibody	Biolegend	Cat# 116235; RRID:AB_11204244
Brilliant Violet 711™ anti-mouse NK-1.1 Antibody	Biolegend	Cat# 108745; RRID:AB_2563286
Brilliant Violet 785™ anti-mouse/human CD11b Antibody	Biolegend	Cat# 101243; RRID:AB_2561373
PE anti-mouse CD179a (VpreB) Antibody	Biolegend	Cat# 143603; RRID:AB_11147372
PE/Dazzle™ 594 anti-mouse CD182 (CXCR2) Antibody	Biolegend	Cat# 149317; RRID:AB_2750072
BD Pharmingen™ PE-Cy™7 Rat Anti-Mouse Ly-6A/E	BD Biosciences	Cat# 561021; RRID:AB_2034021
BD Pharmingen™ Alexa Fluor® 700 Rat Anti-Mouse CD3 Molecular Complex	BD Biosciences	Cat# 561388; RRID:AB_10642588
Brilliant Violet 605™ anti-mouse Ly-6C Antibody	Biolegend	Cat# 128035; RRID:AB_2562352
BD Pharmingen™ PE-Cy™5 Rat Anti-Mouse CD45R/B220	BD Biosciences	Cat#553091; RRID:AB_394621
Donkey anti-Rabbit IgG (H+L) Highly Cross-Adsorbed Secondary Antibody, Alexa Fluor 647	Invitrogen	Cat#A-31573; RRID:AB_2536183
Alexa Fluor 594 Anti-alpha smooth muscle Actin antibody [1A4]	Abcam	Cat#ab202368;RRID:AB_2924381
Anti-PDGFRB antibody produced in rabbit	Merck	Cat# HPA028499; RRID:AB_10602018
Mouse Anti-Human Type IV Collagen-FITC (2F11)	SouthernBiotech	Cat#1460-02; RRID:AB_2794763

**Chemicals, peptides, and recombinant proteins**		

Methyl-beta-cyclodextrin	Sigma	Cat#332615
Hydroxy-L-proline	Sigma	Cat#441562
4%paraformaldehyde(PFA)	Morphisto	Cat.#11762.05000
Tetrahydrofuran	Sigma	Cat#186562
Benzyl alcohol	Sigma	Cat#24122
Benzyl benzoate	Sigma	Cat#W213802
Ethylenediamine tetra acetic acid	Carl Roth	Cat#1702922685
CHAPS	Roth	Cat# 1479.4
Guanidine hydrochloride	Roth	Cat# 6069.3
Acetic acid	Roth	Cat# T179.1
TritonX-100	PanReac Applichem	Cat.#A4975,1000
N-Methyldiethanolamine	Sigma	Cat.#471828
Dichloromethane	Roth	Cat.#KK47.1
RPE buffer	Qiagen	Cat#1018013
Propidium iodide	ThermoFisher	Cat.#P3566
Lycopersicon Esculentum (Tomato) Lectin	Invitrogen	Cat.# L32470

**Critical commercial assays**		

10x Chromium Single Cell 3′ Library & Gel Bead Kit v3 for mouse and v3.1 for human	10x Genomics	https://www.10xgenomics.com/support/single-cell-gene-expression
RNAscope Multiplex Fluorescent Reagent Kit v2 Assay	Advanced cell Diagnostics, Bio-Techne	https://acdbio.com/rnascope-multiplex-fluorescent-v2-assay

**Deposited data**		

Mass spectrometry raw data	This paper	PRIDE accession code: PXD041665
All code used in this study	This paper	https://github.com/erturklab/skull_immune
Patient source file	This paper	Table S3
Single-cell sequencing raw counts matrices and annotation and bulk RNA-sequencing data	This paper	NCBI’s GEO: GSE192616

**Experimental models: Organisms/strains**		

*LySM-GFP* (Lyz2tm1.1^Graf^, MGI: 2654931)	MMRC	Strain#012039-MU;RRID: MMRRC_012039-MU
5xFAD (B6SJL-Tg(APPSwFlLon,PSEN1^∗^M146L^∗^L286V)6799Vas/Mmjax MGI:3693208)	Jackson Laboratory	Strain#034848-JAX;RRID: MMRRC_034848-JAX
KikGR33 (Tg(CAG-KikGR)33Hadj/J)	Jackson Laboratory	Strain#013753:RRID:IMSR_JAX:013753
C57BL/6J mouseline	Jackson Laboratory	Strain#:000,664;RRID:IMSR_JAX:000,664
C57BL/6NJ mouseline	Jackson Laboratory	Strain#:005304:RRID:IMSR_JAX:005304

**Software and algorithms**		

ImSpector	MiltenyiBiotec	https://www.miltenyibiotec.com/DE-en/products/ultramicroscope-blaze.html
Imaris	Bitplane AG	https://imaris.oxinst.com/
Vision4D	Arivis	https://www.arivis.com/de/
Fiji	Schindelin et al.^82^	https://ImageJ.net/software/fiji/
syGlass VR	syGlass	https://www.syglass.io
Scanpy v. 1. 6	Wolf et al.^83^	https://scanpy.readthedocs.io/en/stable/
GraphPadPrism (8.2.1)	GraphPad software	https://www.graphpad.com
PMOD	Digilent	https://www.pmod.com/web/
IBM SPSS Statistics version 22.0	IBM	https://www.ibm.com/spss
Image analysis algorithm	This paper	https://github.com/erturklab/skull_immune

**Other**		

0.22 μm syringe filter	Sartorius	Cat#16532
SCEM medium	Sectionlab	SCEM
70 μm Falcon™ Cell Strainers	Falcon	Cat#08-771-2
35° ultra-diamond knife	Diatome	https://www.scienceservices.eu/tools-supplies/diamond-knifes/ultra
EconoSpin(TM) All-in-1 Mini Spin Clumns for DNA/RNA extraction	Epoch life sciences	Cat#1920-250

### Resource availability

#### Lead contact

Further information and requests for resources should be directed to and will be fulfilled by the lead contact, Ali Erturk (ali.erturk@helmholtz-munich.de).

#### Materials availability

This study did not generate new unique reagents.

### Experimental model and study participant details

#### Animals

Animal housing and experiments in this work were conducted in agreement with the institutional guidelines (Klinikum der Universität München/Ludwig Maximilian University of Munich, Technische Universitaet Muenchen, Regierung von Oberbayern and UK Home Office), after approval of the ethical review board of the government of Upper Bavaria (Regierung von Oberbayern, Munich, Germany), and in accordance with the European directive 2010/63/EU for animal research. The transgenic lines used in this study are C57BL/6, LySM-GFP (Lyz2tm1.1Graf, MGI: 2654931) and 5xFAD (B6SJL-Tg(APPSwFlLon,PSEN1^∗^M146L^∗^L286V)6799Vas/Mmjax MGI:3693208) acquired from Charles River and Jackson Laboratory. KikGR33 (Tg(CAG-KikGR)33Hadj/J) mice were kindly given to C.B. by Dr. Josef Anrather, Weill Cornell Medical College, New York. KikGR33 mice were bred and housed at the animal core facility of the Center for Stroke and Dementia Research (Munich, Germany), and for which male mice were used. 3-month-old male mice were used in study with the exception of bulk AD and 5xFAD TSPO-PET dataset, where the sex of the animals were female and the age were 6-months-old for bulk AD and 4.5-months-old for TSPO-PET dataset. In all in vivo experiments in this study, littermates of the same sex were randomly assigned to experimental groups, the animals were housed under a 12/12 h light/dark cycle, all animals were healthy in the beginning of the experiment, no drugs were given to animals during the time of data acquisition, no specific food, temperature and cage conditions were kept. All data are reported according to the ARRIVE.^84^

#### Human samples and participants

All anatomy donors or next-of-kin gave their informed and written consent to explore the cadavers for research and educational purposes. The signed consents are kept at the institutes involved. Institutional approval was obtained in accordance to the Saxonian Death and Funeral Act of 1994, of the independent ethics committee of the Hamburg Chamber of Physicians (protocol 2020-10353-BO-ff) and the Ethics committee of Technical University of Munich (67/22S). The skull samples in this study are coming from the following sources: University Medical Center Hamburg-Eppendorf, Institute of Legal Medicine; Institut für Allgemeine Pathologie und Pathologische Anatomie, Technische Universität München and Anatomy Institute, University of Leipzig, Institut für Rechtsmedizin der Universität München. A detailed list of post-mortem samples used for light sheet imaging and proteomics samples are given in Figures S5O and S7A.

Regarding human participants: ten patients with stroke, 29 patients with multiple sclerosis (15 with relapsing remitting multiple sclerosis and 14 with primary progressive multiple sclerosis), 43 patients with 4R tauopathies, 50 patients with AD and 27 age- and sex-matched individuals without objective cognitive impairment and with intact motor function were available for calvaria analysis of TSPO-PET. Sample sizes were determined in the specific study protocols, based on comparisons of TSPO-PET signals in brain between disease and controls. Power was set to 0.8 and alpha was set to 0.05 with the goal to achieve effect sizes of 1.0, also graded sufficient to test for differences in skull TSPO-PET signals. Allocation into study groups was determined by the clinical diagnosis. Severe neurological disorders other than the investigated diagnosis were excluded in the study protocols, assuming immunocompetence in all participants. All participants were naïve to TSPO-PET at study inclusion. In one set of analyses stroke, MS and 4R tauopathy patients were compared with controls, while the AD cohort, for which additional biomarkers were available, was analyzed separately. All patients with multiple sclerosis were investigated during observational studies. We included all baseline scans of therapy naïve patients with primary progressive multiple sclerosis (n=14) and patients with relapsing remitting multiple sclerosis (n=15; previously published in Unterrainer et al.^63^) regardless of therapy regimes. However, patients who received steroid therapy < 4 weeks prior to PET as well as patients with additional CNS pathologies were excluded a priori. PET acquisition and PET data analyses of the multiple sclerosis cohort (ethics-application: 601–16) were approved by the local institutional ethics committee (LMU Munich) and the German radiation protection (BfS-application: Z 5 – 22463/2 – 2015 - 006) authorities. The 4R-tauopathy cohort^62^ was composed of patients with possible or probable β-amyloid negative corticobasal syndrome (n=29) and patients with possible or probable progressive supranuclear palsy Richardson syndrome (n=14) according to Armstrong Clinical Research and Movement Disorders Society criteria respectively. Detailed inclusion and exclusion criteria were published elsewhere.^62^ One case was excluded due to cropped skull. PET acquisition and PET data analyses of the 4R-tauopathy cohort (ethics-applications: 17–569 & 17–755) were approved by the local institutional ethics committee (LMU Munich) and the German radiation protection (BfS-application: Z 5 - 22464/2017-047-K-G) authorities. A total of 27 healthy controls deriving from the different cohorts were included to cover the whole age range of patients. PET acquisition and PET data analyses of the stroke cohort (ethics-application: 19–428) were approved by the local institutional ethics committee of the LMU Munich (ethics-application: 19–428) and the German radiation protection authority (BfS-application: Z 5 - 22464/2019-163-G). To compare different patient cohorts, we used harmonized data from different PET imaging studies: All patients with acute ischemic stroke (n=10) were recruited from the ongoing ICARUS study which included a TSPO-PET up to 10 days after stroke onset. Inclusion criteria were an age >50 years, acute ischemic stroke as defined by an acute focal neurological deficit in combination with a corresponding infarct as documented by diffusion-weighted imaging (DWI)-positive lesion on brain MRI, presence of an infarct involving the cortex or a strictly subcortical infarct, written informed consent; and willingness to participate in study assessments including follow-up. Exclusion criteria were among others multiple infarcts, infratentorial infarcts affecting the brain stem or cerebellum, immunomodulatory therapies within the last 3 months, chronic inflammatory disease, and infectious diseases (< 7 days prior to stroke). The AD cohort was composed of nine cases with subjective cognitive decline due to AD, 13 cases with mild cognitive impairment due to AD, 18 cases with AD dementia, and 12 cases with corticobasal syndrome, dementia and underlying AD. Initial results of brain TSPO labeling in this cohort are published elsewhere.^85^ Two patients with AD were excluded from the cross-sectional TSPO-PET group comparison due to limited field of view. Participants were enrolled in the interdisciplinary AD study "Activity of Cerebral Networks, Amyloid and Microglia in Aging and AD (ActiGliA)". In the AD cohort and its controls, Aβ-PET was performed in all participants using [^18^F]flutemetamol.^86^ PET acquisition and PET data analyses of the AD cohort (ethics-applications: 17-569 & 17-755) were approved by the local institutional ethics committee (LMU Munich) and the German radiation protection (BfS-application: Z 5 - 22464/2017-047-K-G) authorities. Longitudinal follow-up imaging within the ActiGliA cohort was available for 13 patients of the AD continuum and 15 controls at a median interval of 18 months. Additionally, 3 months follow-up imaging was available for 13 patients with stroke. Age, gender, SNP coding and medication status of participants are provided in Table S3.

### Method details

#### Middle cerebral artery occlusion (MCAo) model

The MCAo model was used to generate transient cerebral ischemic strokes by introducing an intraluminal filament through the carotid artery of mice anesthetized with isoflurane mixed with 30% O_2_ and 70% N_2_O. To initiate the occlusion the left common carotid artery and interna of the animal were permanently ligated and a silicon capped nylon suture (6/0) was introduced through a cut in the common carotid artery and advanced through the external carotid artery until it reached and obstructed the MCA for 30 minutes. Regional cerebral blood flow was monitored, in the bregma coordinates 2-mm posterior, 5-mm lateral, via transcranial laser Doppler flowmeter from the induction of stroke until 10 minutes after retraction of the filament and reperfusion took place. After the procedure, mice were left for recovery in temperature-controlled cages for two hours in order to minimize the risk of hypothermia. Sham-operated animals were subjected to the same procedure without the insertion of the filament. Body temperatures were kept constant throughout all surgeries with a feedback-controlled heating pad at 37.0 ± 0.5 °C. Animals were then kept in their home cages with facilitated access to water and food whilst being subjected to behavioral tests for three days. Mice were excluded in case of insufficient MCA occlusion (a reduction in blood flow to 15% of the baseline value) or blood flow recovery >80% within 10 min of reperfusion.

#### Skull preparation for chronic imaging

Experiments were carried out on 8-12 weeks old male LysM-GFP -/+ mice. Induction of anesthesia with buprenorphine (0.1 mg/kg Bw) and isoflurane (5%, 30s), was followed by maintenance anesthesia with 1.5-2.5% isoflurane in room air with 30% oxygen/70% air under continuous monitoring of body temperature 37.5 C° with a feedback-controlled heating pad. Glass window preparation was adapted from described method.^87^ Mouse was placed on a stereotactic frame (RWD Life Science Co.,LTD, Shenzhen, China) where head was fixed by ear bars and the eyes were covered with Bepanthen ointment (AG Bayer, Leverkusen, Germany). The left parietal bone was exposed after resection of the mouse scalp. Sterile saline was applied to the skull and the periosteum were gently removed with forceps. Then, Ultraviolet-curable glue Loctite 4305 (Henkel, Düsseldorf, Germany) was applied onto the parietal bone surface. A sterile round glass window of 3-mm diameter was placed on the skull followed by two by 1s exposures every 3s of ultraviolet light source UV301D UV 365NM Light Flashlight (LIGHTFE Lighting Co., Ltd., Shenzhen, China) to cure the glue. Then, skull surrounding the window was prepared for the dental cement application by putting for 1 min onto the surface of iBond Self Etch (Kulzer GmbH., Hanau, Germany) solution with subsequent curing by UV source for 5 s using Demi™ Ultra Dental Curing Lights (Kerr Corporation, Brea, CA, USA). Then on top of the etching solution the dental cement Tetric EvoFlow® (Ivoclar Vivadent, Schaan, Liechtenstein) was applied. Before UV curing, the titanium ring was placed on the skull to have the window in its center and then cement was cured with the same UV source (Demi™ Ultra Dental Curing Lights) for not more than 5 s including every side around the ring. Finally, a thin stripe of dental cement was applied onto the inner edge of the ring, with subsequent 5 s UV exposing, to fix the ring to the skull. Carprofen (4mg/kg every 24h) was administered i.p. for the following 72 hours.

#### Live Imaging

For multiphoton imaging, we used an upright Zeiss LSM710 confocal microscope equipped with a Ti:Sa laser (Chameleon Vision II) from Coherent (Glasgow, Scotland) and 2 external photomultiplier detectors for red and green fluorescence.^88^ Anesthetized animals (1.5 % of isoflurane) were placed on a heating pad under the microscope. For visualization of the vasculature, 5 min prior to the imaging, the fluorescent tracer Tetramethylrhodaminisothiocyanat-Dextran (TMR-Dextran), 3000 Da MW (Sigma-Aldrich, St.Luis, MI, UA) was injected subcutaneously. The scanning was performed with Z-stack, 50-100 μm depth, laser (900 nm) power from 6-8% till 12-16% depending on the region of interest (ROI) depth. GAASP detector with LP˂570 nm filter for the GFP channel, LP˃570 nm for the TMR channel, and NDD detector SP˂485 nm for the bone visualization, all with master gain 600. Image size 1024x1024, 8 bit. Objective: W Plan-Apochromat 20x/1.0 DIC M27 75mm. For the series scanning, the laser power was 8-10%, 5 frames every 1 s. For each animal, 2-3 ROI was chosen which were imaged at baseline, 2, 24, and 72 hours post-stroke, or at the respective time point for naïve and sham-operated animals.

#### Behavioral experiments - Neuroscore

Neuroscore^24^ was performed to assess each animal's general and focal deficits every day. The scoring was composed of general deficits (scores): fur (0 to 2), ears (0 to 2), eyes (0 to 4), posture (0 to 4), spontaneous activity (0 to 4), and epileptic behavior (0 to 12); and focal deficits: body asymmetry (0 to 4), gait (0 to 4), climbing on a surface inclined at 45° (0 to 4), circling behavior (0 to 4), fore-limb asymmetry (0 to 4), compulsory circling behavior (0 to 4), and whisker response to touch (0 to 4). This resulted in a score of 0 to 56 in total; up to 28 from general and up to 28 from focal deficits.

#### Perfusion, fixation and tissue preparation

After the mice were anesthetized with a mixture of midazolam, medetomidine and fentanyl (MMF) (1ml/100g of body mass for mice; i.p.), and showed no pedal reflex, they were intracardially perfused with 0.1 M PBS (combined with heparin, 10 U/ml, Ratiopharm). 100-125 mmHg pressure with a Leica 13 Perfusion One system was used for perfusion. PBS ran for 3-4 minutes for single-cell isolation experiment, 5-10 minutes for tissue clearing experiments to let the blood wash out at room temperature. For single-cell isolation experiments, bones were dissected as detailed in the Single cell isolation method section. For the tissue clearing experiments, PBS perfusion was followed by the administration of 4% paraformaldehyde (PFA) in 0.1 M PBS (pH 7.4) (Morphisto, 11762.01000) for 10-20 minutes. After removal of the skin and a washing step with PBS to clean the animal as much as possible, the animals were post-fixed by 4% PFA for the first 24 hours at 4°C and washed three times with 0.1M PBS before processing with the clearing protocol.

#### vDISCO whole-body immunostaining, PI labeling and clearing

The detailed protocol of vDISCO was described previously.^2^^,^^89^ The mouse bodies were placed inside a 300 ml glass chamber (Omnilab, 5163279), to be filled with the appropriate solution regarding the protocol to cover the entire body of the animal (∼250-300ml). A transcardial-circulator system was established in order to allow peristaltic pumping of the solutions (ISMATEC, REGLO Digital MS-4/8 ISM 834; reference tubing, SC0266), with the pressure being set at 180-230 mmHg (50-60 rpm). The tubing was set to allow pumping of the solutions through the heart (attached to a perfusion needle (Leica, 39471024)) into the vasculature with the same entry point used for PBS and PFA perfusion steps described above. The other end of the tube was immersed into the chamber with a loose end to allow suction of the solution into the body. The samples were initially perfused with a decolorization solution (25% of CUBIC reagent 1^90^ which is composed of 25 wt% urea (Carl Roth, 3941.3), 25 wt% N,N,N',N'-tetrakis (2-hydroxypropyl)ethylenediamine (Sigma, 122262) and 15 wt% Triton X-100 (AppliChem, A4975,1000) in 0.1 M PBS)) for 2 days, refreshing the solutions every 12h. Samples were washed with PBS for 3x2h. Then, decalcification solution (10 wt/vol% EDTA in 0.01 PBS, pH∼8-9, Carl Roth, 1702922685) was perfused for 2 days followed by half a day with permeabilization solution composed of 0.5% Triton X-100, 1.5% goat serum (GIBCO, 16210072), 0.5 mM of Methyl-beta-cyclodextrin (Sigma, 332615), 0.2% trans-1-Acetyl-4-hydroxy-L-proline (Sigma, 441562), 0.05% sodium azide (Sigma, 71290) in 0.01 M PBS. To initiate the PI labeling and boosting, the setup was adjusted. The free end of the perfusion tube was connected to a 0.22 μm syringe filter (Sartorius, 16532) and an infrared lamp (Beuer, IL21) was aimed at the chamber to enable the solution's temperature to be around 26-28 °C. This setup was then left running for 6 days after the addition of 35 μl of nanobooster (stock concentration 0.5 – 1 mg/ml) and 290 μl of propidium iodide (stock concentration 1 mg/ml) which was added directly into the refreshed permeabilization solution. Next, the body was placed into a 50 ml tube (Falcon, 352070), with the same permeabilization and labeling solution, and an extra 5 μl of nanobooster was added. The tube was then put on a shaker at RT for 2 additional days for labeling. Atto647N conjugated anti GFP nanobooster (Chromotek, gba647n-100) and Propidium iodide (PI, Sigma, P4864), was used to boost the signal from the LysM animals and stain cell nuclei respectively in the study. Then, the animals were placed back into the initial perfusion setup, where the washing solution was perfused for 2x12h, which was composed of; 1.5% goat serum, 0.5% Triton X-100, 0.05% of sodium azide in 0.1 M PBS. 0.1 M PBS was used to wash the sample 3x2h. 3DISCO protocol was applied for whole body clearing. The animals were freed from the perfusion system, but kept in glass chambers and placed on top of shakers (IKA, 2D digital) at room temperature inside a fume hood. Glass chambers were sealed with parafilm and covered with aluminum foil along with the 3DISCO application. For dehydration, sequential immersion of tetrahydrofuran (THF) (Sigma, 186562) (50 Vol% THF, 70 Vol% THF, 80 Vol% THF, 100 Vol% THF and again 100 Vol% THF ) was applied every 12 hours. Then three hours of dichloromethane (DCM) (Sigma, 270997) immersion for delipidation was followed by indefinite immersion in BABB (benzyl alcohol + benzyl benzoate 1:2, Sigma, 24122 and W213802) solution for refractive index matching.

#### *Nr4a1* labeling and clearing of mice bones with SHANEL

Mouse heads and left femurs were collected from three-month-old, male, wild-type C57Bl6/J mice (n=3). After dissection to remove surrounding tissue, bones were decalcified in 20% (wt/vol) ethylenediamine tetraacetic acid (EDTA, pH=8.0, prepared in dH2O) for two days at 37°C. EDTA was removed by washing in 0.1 M PBS for 3x2 hours. Bones were stained and cleared using the SHANEL protocol.^91^ Samples were dehydrated in 50–70–100% ethanol/dH2O for 1 hour each, delipidated in dichloromethane/methanol (2:1) for 6 hours and rehydrated in sequence with the same dilutions backward. Next, they were incubated in acetic acid/dH2O (30 mL/L) 2 hours for extracellular matrix hydrolyzation and washed with dH2O for 3x15 minutes. Then, samples were incubated in extracellular matrix proteoglycan extraction solutions consist of 4 M guanidine hydrochloride, 0.05 M sodium acetate, and 2% Triton X-100 in 0.1 M PBS for 2 hours, washed first with dH2O and then PBS for 3x15 minutes each, followed by incubation in 10% 3-[(3-Cholamidopropyl)-dimethylammonio]-1-propansulfonat (CHAPS) + 25% N-Methyldiethanolamine (NMDEA) in dH2O at 37°C for further permeabilization and washed with dH2O for 3x15 minutes. Blocking was performed with 0.2% TritonX-100, 10% DMSO, 10% goat serum in 0.1 M PBS (blocking solution) for 6 h at 37°C. Anti-NUR77 antibody (1:200, Abcam, ab153914) were added with 0.2% Tween-20, 5% dimethyl sulphoxide (DMSO), 5% goat serum, 0.001% heparin in 0.1 M PBS (primary antibody solution) and incubated for 2 days at 37°C. After washing with 0.2% Tween-20, 0.001% heparin in 0.1 M PBS (washing solution) 4x20 minutes, bones were incubated with Goat anti-Rabbit IgG (H+L) Highly Cross-Adsorbed Secondary Antibody, Alexa Fluor 647 (1:200, Abcam, A-21245) and propidium iodide (1:1000) in 0.2% Tween-20, 5% goat serum, 0.001% heparin in 0.1 M PBS (secondary antibody solution) for 2 days at 37°C, washed with washing solution. Clearing was performed by dehydrating the bones in dilutions of 50-70-100-100% ethanol/dH2O for 12 h each, followed by delipidation of the samples in 100% dichloromethane (DCM) for 15 minutes. Finally, samples were kept in refractive index matching solution BABB (benzyl alcohol + benzyl benzoate 1:2, Sigma, 24122 and W213802). If not stated otherwise, steps were performed with constant shaking at room temperature inside a fume hood.

#### Human skull labeling and clearing with SHANEL

SHANEL protocol with extended incubation periods were performed on human post-mortem skull pieces. Bones were decalcified in 20% (EDTA, pH=8.0) for 1.5-2 months (until the bone becomes cuttable) at 37°C EDTA was removed by washing in 0.1 M PBS for 3x2 hours. Bones were incubated in 10% CHAPS + 25% NMDEA solution for 4 days at 37°C and washed with dH2O for 3x20 minutes. Bones were dehydrated in 50-70-100% ethanol/dH2O, each with overnight incubation, delipidated overnight in DCM/MeOH (2:1) and rehydrated. Then, samples were incubated in acetic acid for 4 hours, followed by incubation with 4 M guanidine hydrochloride, 0.05 M sodium acetate, and 2% Triton X-100 in 0.1 M PBS for 2 days and washed first with dH2O and then PBS for 3x20 minutes. Samples were incubated in a blocking solution overnight at 37°C. Staining was performed in two groups: some samples were incubated with recombinant anti-Lysozyme antibody (1:250, Abcam, ab108508) in primary antibody solution for 10 days at 37°C, washed with washing solution 4x20 minutes, then stained with Lycopersicon Esculentum (Tomato) Lectin (LEL, TL), DyLight 649 (1:500, Invitrogen, L32470) for 7 days at 37°C, washed with washing solution 4x20 minutes, incubated with Goat anti-Rabbit IgG (H+L) Highly Cross-Adsorbed Secondary Antibody, Alexa Fluor 568 (1:200, A-11036, Thermo Fisher) and washed. The remaining samples were stained with Lycopersicon Esculentum (Tomato) Lectin and Propidium Iodide (1:1000, Sigma, P4864) in primary antibody solution, washed and proceeded with clearing. Clearing was performed by dehydrating the bones in dilutions of 50-70-100-100% ethanol/dH2O for 12 h each, delipidated in 100% DCM for 15 minutes and incubated indefinitely in BABB (benzyl alcohol + benzyl benzoate 1:2, Sigma, 24122 and W213802). If not stated otherwise, steps were performed with constant shaking at room temperature inside a fume hood.

#### Light sheet microscopy imaging

Single plane illumination (light sheet) image stacks were acquired using an Ultramicroscope II and Ultramicroscope Blaze (Miltenyi BioTec). The available filter sets were ex 470/40 nm, em 535/50 nm; ex 545/25 nm, em 605/70 nm; ex 560/30 nm, em 609/54 nm; ex 580/25 nm, em 625/30 nm; ex 640/40 nm, em 690/50 nm. The filter sets used to capture the LysM signal and the PI labeling were 640/40 nm and 545/25 nm filter sets, respectively. Low magnification whole-body imaging of the LysM mice was performed with Ultramicroscope Blaze, with a 1.1x objective, 3x8 tiling with 35% overlap and 6 μm z-step. Exposure time was 120 ms, and laser power was 25% and 12% for LysM (647nm) and PI (594nm) channels, respectively. The depth of the scans was approximately 13 mm from dorsal and ventral surfaces, which were then reconstructed. The whole head images were taken with an Olympus MVX10 zoom body, which offered zoom-out and -in ranging from 0.63x up to 6.3x. The depth of the scans was approximately 4 mm and the z-step used was 6 μm combined with an exposure time of 200 ms. Human bone pieces were imaged with 1.1x magnification using LaVision BioTec MI PLAN 1.1x/0.1 NA (WD = 17 mm), with 1.66X zoom, as stacks, tiles were adjusted to cover all the bone surface with 25-35% overlap. The depth of scans was 1-1.5 cm. Higher magnification imaging of ROIs from mice bones were obtained with 12x magnification using PLAN 12x/0.53 NA (WD = 10 mm), LaVision 470 BioTec MI) objective as 1 tile, step size of 6 μm. Depth of scans was 0.5-1 mm. Following settings were kept the same for all samples: Exposure time: 120 ms; light sheet width: 100%; and light sheet thickness: 7 μm (NA 0.31). Multiple tile scans were stitched with Fiji’s stitching plugin (http://discotechnologies.org/SHANEL/manual_stitching.py) and visualized in 3D using Imaris (v.9.6 × 64, Imaris).

#### Reconstruction of whole-mouse body and mouse head scans

The image stacks were acquired and saved by ImSpector (Miltenyi BioTec) as 16-bit grayscale TIFF images for each channel separately. The whole-body tiled stacks were initially stitched utilizing Fiji/ImageJ to obtain stitching on the xy-axis (http://discotechnologies.org/SHANEL/manual_stitching.py). Next, Vision 4d (Arivis AG) was used to fuse the stacks in the z-axis. For heads, one tile stacks were acquired, hence stitching was not necessary. Imaris (Bitplane AG) was used to visualize both whole body and intact mouse heads.

#### Fixed-formalin paraffin embedding, sectioning and H&E staining

Fixed formalin paraffin-embedded samples were acquired from decalcified human skulls initially by using the Sakura Tissue-Tek VIP 6 AI machine. The samples were placed in holders and were sectioned as 1 μm thick slices using Microm HM 355S microtome. Sections were then placed in the Sakura Tissue-Tek Prisma machine for H&E staining. The images were then acquired by a bright field microscope using 40x magnification.

#### Human fixed frozen sections, immunohistochemistry and imaging

Fixed samples were placed in 15% sucrose in PBS until they sunk and then in 30% sucrose overnight. Samples were frozen in SCEM medium (Sectionlab, Japan). 7 μm cryosections were cut using Kawamoto‘s film method^92^ on a cryostat, which were then stored at −80°C until further use. For immunofluorescence, sections were thawed, rehydrated in PBS, blocked with 10% serum, and stained with antibodies in 1% serum in PBS containing DAPI for 1–2 h. Antibodies used were; anti-SMA-Alexa Fluor A594, 1A4, Abcam; Collagen-IV-FITC, 2F11, Southern Biotec; anti-PDGFRB, HPA028499, Merck; Donkey anti-Rabbit IgG (H+L) Highly Cross-Adsorbed Secondary Antibody, Alexa Fluor 647. Stained sections were washed and mounted with aqueous mounting medium (Fluoromount, Thermo Fisher, MA, US).

Sections were imaged with at a Zeiss LSM880 using a 20x objective.

#### Single-cell isolation for scRNAseq and proteomics

Single-cell isolation from the calvaria, brain, meninges, humerus, scapula, vertebra, femur and pelvis was done for one animal at a time. Three naïve, six MCAo-operated and three sham operated animals were pooled in threes for single-cell RNA sequencing. Another cohort of three animals for naïve, three animals for sham-operated and three MCAo-operated animals were not pooled and were treated separately for proteomic analysis. These experiments were performed on sham and MCAo animals that had the procedure three days prior to the single-cell isolation experiment. Separate equipment was utilized during the isolation to ensure high viability of cells free of contamination. The animals were anesthetized with Isoflurane and then with a Ketamine/Xylazine mixture (0.6 ml Ketamine + 0.3 ml Xylazine + 5.1 ml Saline, 0.2 ml for 20 gr animals). Then animals were transcardially perfused with 10 ml of ice-cold 0.1 M PBS. After the blood was rinsed, the calvaria bone, humerus, scapula, vertebra, femur, brain, meninges, and pelvis were dissected and processed by separate people to minimize the time required in order to keep the cell viability to a maximum and conditions comparable for all locations. The isolated cells were processed with 37°C pre-warmed DMEM (Thermo Fischer, 21013024) with 10% heat inactivated fetal bovine serum (FBS) (Sigma Aldrich, F7524-100ML). For brain cell isolation; the brain was isolated from the calvaria and the rest of the body, then, the cortex was separated and the leptomeninges was removed from the surface, the final sample consisted of the injured region. The sample was placed in 5 ml of trypsin enzyme with 0.05% concentration and incubated in a pre-heated 37°C water bath for 2 minutes. Following this, the reaction was stopped with 10 ml of 37°C pre-warmed DMEM with 10% heat inactivated FBS, the cells were dissociated by gentle tritration with a 1000μl and 200μl pipette and filtered through 70 μm Falcon™ Cell Strainers (08-771-2). For meningeal cell isolation; after the brain was removed, the meningeal dura layer that was attached to the calvaria bone, was plucked carefully using fine tipped dissection pincers (Dumont #55 Forceps, Dumostar, 11295-51, FST) under a dissection microscope. Leptomeninges was not isolated and therefore is not included in this study. The dissected meninges was placed in 37°C pre-warmed DMEM with 10% heat-inactivated FBS solution, shredded with a fine scalpel, gently titrated with a 200 μl pipette and filtered through a 70 μm Falcon™ Cell Strainers (08-771-2). For humerus, vertebrae and femur cell isolation; the bone was dissected from the body and the muscles and connective tissue were meticulously cleared off. The bone marrow inside was flushed out to the collection tube with the help of a syringe (Braun, Injekt - F Solo 2-piece Fine Dosage Syringe 1 ml x 100), and further dissection of the bone was performed by fine pincers (Dumont #55 Forceps, Dumostar, 11295-51, FST). The remaining bone was cut into small pieces and added to the cell mix. This mixture was shortly vortexed with 37 °C warmed DMEM with 10% heat-inactivated FBS and filtered with 70 μm (Falcon™ Cell Strainers, 08- 771-2). Lastly, for the flat bones, calvaria, scapula and pelvis, after carefully clearing the non-bone parts in the sample i.e., muscles and connective tissue, they were cut into small pieces (Extra Fine Bonn Scissors, 14084-08, FST), and shortly vortexed and filtered through 70 μm Falcon™ Cell Strainers (08-771-2). After all the samples were ready, they were centrifuged at 4 °C, with 1000 rpm, for 5 minutes. The supernatant of all samples was then discarded and the remaining precipitate was put into small 1.5 ml Eppendorf tubes (Eppendorf Safe-Lock Tubes, 1.5 mL, Eppendorf Quality™, 0030120086) after resuspension with DMEM. Cell viabilities and numbers were checked with trypan blue by an automated cell counter (TC20™ Automated Cell Counter) and controlled by manual counting (Neubauer Cytometry Chamber, MARI0640031).

#### Cell sorting and plate-based bulk RNA-sequencing

6-month-old mice were used for this study (3 5XFAD, 3 wildtype littermates). Cell sorting for CD45 and CD11b positive cells, cDNA generation and library construction was performed as described previously.^93^ Briefly, after cells were passed through a 70 μm cell strainer, staining was performed for 15 min using 7AAD (Thermo Fisher, A1310, 25 ug/ml) and the antibodies against CD45 (eFluor 450,30-F11, eBioscience,Cat.:48-0451-82, 1:200) and CD11b (PE/Cy7,M1/70, eBioscience, Cat:25-0112-82,1:200). Cells were then washed with PBS (Sigma, D8537). Viable (7AAD negative) immune cells (CD45 and CD11b positive cells) were sorted by flow cytometry (SH800; Sony) into the 96-well plates by groups of 50 cells per well (we acquired 69 samples). The 96-well plates were filled with 4 μl lysis buffer containing 0.05% Triton X-100 (Sigma), ERCC (External RNA Controls Consortium) RNA spike-in Mix (Ambion,Life Technologies) (1:24000000 dilution), 2.5 μM oligo-dT, 2.5 mM dNTP and 2 U/μl of recombinant RNase inhibitor (Clontech). The plate was spun down and frozen at -80 C.

cDNA and cDNA libraries were generated using an improved version of the Smart-seq2 protocol. The plates with the sorted pools were first thawed and then incubated for 3 min at 72°C and immediately placed on ice. To perform reverse transcription (RT), we added to each well a mix of 0.59 μl H2O, 0.5 μL SMARTScribe™ Reverse Transcriptase (Clontech), 2 μL 5x First Strand buffer, 0.25 μl Recombinant RNase Inhibitor (Clontech), 2 μl Betaine (5 M Sigma), 0.5 μl DTT (100 mM), 0.06 μl MgCl2 (1 M Sigma), and 0.1 μl Template-switching oligos (TSO) (100 μM AAGCAGTGGTATCAACGCAGAGTACrGrG+G). Next, RT reaction mixes were incubated at 42°C for 90 min followed by 70°C for 5 min and 10 cycles of 50°C 2 min, 42°C 2 min; finally ending with 70°C for 5 min for enzyme inactivation. Pre-amplification of cDNA was performed by adding 12.5 μl KAPA HiFi Hotstart 2x (KAPA Biosystems), 2.138 μl H2O, 0.25 μl ISPCR primers (10 μM, 5′ AAGCAGTGGTATCAACGCAGAGT-3), 0.1125 μl Lambda Exonuclease under the following conditions: 37°C for 30 min, 95°C for 3 min, 20 cycles of (98°C for 20 sec, 67°C for 15 sec, 72°C for 4 min), and a final extension at 72°C for 5 min. Libraries were then cleaned using AMPure bead (Beckman-Coulter) cleanup at a 0.7:1 ratio of beads to PCR product. Library was assessed by Bioanalyzer (Agilent 2100), using the High Sensitivity DNA analysis kit, and also fluorometrically using Qubit’s DNA HS assay kits and a Qubit 4.0 Fluorometer (Invitrogen, LifeTechnologies) to measure the concentrations. Samples were normalized to 160 pg/μL. Sequencing libraries were constructed by using an in-house produced Tn5 transposase.^94^ Libraries were barcoded with the Illumina Nextera XT (FC-131-1096, Illumina) and pooled, then went through three rounds of AMPure bead (Beckman-Coulter) cleanup at a 0.8:1 ratio of beads to library. Libraries were sequenced 2x100 reads base-pairs (bp) paired-end on Illumina HiSeq4000.

#### Single-cell suspension isolation from the human bones for proteomics

Bone samples were collected into formalin and were washed with PBS within 24 hours of fixation. Then, samples were placed in 20% EDTA (pH∼8) in 37°C. EDTA was changed every second day. When all bones reached a cuttable softness with scissors and a scalpel, the samples were washed with PBS overnight. 20 skull, vertebra and pelvis were dissected by carefully clearing the non-bone parts in the sample i.e., muscles and connective tissue. The same sizes of bones were cut into small pieces on a glass petri-dish with PBS. The resulting cell suspension was filtered through 40 μm Falcon™ Cell Strainers into a 50 ml Falcon tube. The samples were centrifuged for 5 minutes in 12000g. The supernatant was discarded. The pellet was resuspended in 1 ml pbs and transferred to 1.5 ml Eppendorf tube (Eppendorf Safe-Lock Tubes, 1.5 mL, Eppendorf Quality™, 0030120086). The tubes were centrifuged for another 5 minutes in 12000g. PBS was discarded and samples were stored in -80 until all samples were acquired.

#### Single-cell isolation from human skull for scRNAseq

Sample was sectioned in the clinic with an electric saw to generate thinner, smaller pieces and was collected in DMEM + 10% FCS. The sample was brought to lab from the clinic on ice. Using a needle and a syringe, the bone marrow cells were flushed DMEM + 10% FCS (Gluc + /Glut +) into a 50 ml tube. Bone was further crushed using a mortar on ice in order to release more marrow cells into the cell suspension. Each sample was filtered through a 70 μm strainer and centrifuged at 500 rcf for 5 mins at 4°C. Supernatant was discarded. The pellet was resuspended in 10 ml chilled PBS / 2%BSA. Then, the cells were visually counted using trypan blue to assess the high viability. Next, the samples were washed again as above and resuspended for loading to the 10X Chromium.

#### scRNA sequencing – 10x Genomics

Samples were used for scRNA-seq if the fraction of dead cells determined by trypan blue staining was below 20%. Cell suspensions were diluted with PBS/2% FCS for mouse experiments and PBS/2% BSA for the human experiment, to a final concentration of 1000 cell/μl and 17.000 cells per sample were loaded onto 10x Chromium Single Cell RNA-seq chips to recover a target cell number of 10.000 cells per sample. Libraries were generated in three replicates for the mouse experiment. The 10x Chromium Single Cell 3′ Library & Gel Bead Kit v3 for mouse and v3.1 for human was used following the manufacturer's protocol. Libraries were sequenced on an Illumina HiSeq 4000 (150 bp, paired end) for mouse experiment and NovaSeq6000 for human experiment.

#### Sample preparation for bulk RNA isolation

Mice were deeply anaesthetized with ketamine (120mg/kg) and xylazine (16mg/kg) and transcardiacally perfused with cold PBS. The necessary samples were quickly harvested and placed in ice-cold RNAlater solution in 1.5 ml tubes.^95^ The samples were left in RNAlater solution for 24h in 4 °C. Next, the solution was discarded and samples were placed in -80 for storage until RNA isolation.

#### Bulk RNA isolation, library preparation and sequencing

The samples were processed 12 at a time. Samples were in a 2 ml Eppendorf tube. 1 ml of Trizol and a metal bead was added to each sample. Samples were then lysed in Tissue Lyser with 30 Hz frequency for 3 minutes. 200 μl of Chloroform was added to each sample. After rigorous vortexing, samples were incubated at room temperature for 15 minutes. Next, samples were centrifuged at 10,000 g for 10 minutes at 4°C. The upper phase of the sample was transferred into a new tube. 240 μl, 100% seq-grade EtOH was added and samples were briefly vortexed. The samples were loaded into Econospin columns and were centrifuged at 13000 g for 30 seconds at room temperature. Flow-through was discarded. The samples were washed 3x with RPE buffer (Qiagen #1018013) and centrifuged at 13000 g for 30 seconds each time. After the last wash, sample was centrifuged dry. Next, columns were transferred into new 1.5 ml tubes with open lids for 10 minutes. RNAse free water was used to elute the sample in 30 μl. Final centrifuge was performed at 9000 g for 2 minutes at room temperature. All samples were subjected to Nanodrop, Qubit and Bioanalyzer assays in order to determine quality and quantity of each sample. Samples were stored at -80 until all samples were processed. Only samples with RIN>8 were used. Illumina ligation stranded mRNA prep kit was used for library preparation and the sequencing of 95 samples were performed on a PE 2x100 flow cell.

#### Flow cytometry

##### Cell isolation and labeling

Cell isolations were prepared as previously described in “Single cell isolation for scRNAseq and proteomics” section. The suspended cells were centrifuged at 500g for 7 minutes at 4°C. The supernatant was discarded, and samples were resuspended in 1 ml FACS buffer. The 1 ml buffer with cells was transferred to a FACS tube. Tubes were spun down at 500 g 7 minutes at 4°C. The supernatant was discarded and each sample was resuspended in 50μl FACS buffer with 0.5 μl FC blocker. The samples were incubated for 10 minutes, in dark on ice. Then 50 μl of antibody mix was added to each sample: (1 μl each from Ly-6G/Ly-6C (APC/Cyanine7, Biolegend, #108423), Cd11c (APC, Biolegend #117309), F4/80 (PerCP-Cy5, BD, #567202), CD45 (BUV395, BD. #565967), CD117(BV421, BD, #566290), I-A/I-E (Brilliant Violet 510, Biolegend, #107635), Ter-119 (BV650, Biolegend, #128035), NK-1.1 (Brilliant Violet 711, Biolegend, #108745), CD11b (Brilliant Violet, Biolegend, #101243), CD179a (PE, Biolegend, #143603), CD45R/B220 (PE-Cy5, BD, #553091), CD182 (CXCR2) (PE/Dazzle 594, Biolegend, 149317), Ly-6A/E (PE Cy 7, BD, #561021) and 1.5 μl each from CD3 Alexa Fluor 700, BD, #561388), Ly-6C (Brilliant Violet 605, Biolegend, 128035), 34 μl FACS buffer). Samples were incubated for 15 minutes on ice in dark. After the staining 3 ml of FACS buffer was added to each sample. The samples were centrifuged at 500 g 7 minutes at 4°C. After discarding the supernatant, samples were resuspended in 200 μl of FACS buffer to be measured by the machine.

##### Proportions

Sample were recorded on a LSRFortessa (BD) and data were analyzed with FLowJo software (Tree Star). Cell numbers were calculated as percentage of an appropriate gate. After gating out doublets, dead cells (SytoxGreen+) and red blood cells (Ter119+), white blood cell (CD45+) subpopulations were defined as follows: T-cells (CD3+), immature B-cells (B220dim I-A/I-Evar), mature B-cells (B220+ I-A/I-E+), NK-cells (NK1.1+), monocytes (F4/80+), eosinophils (Ly6G-, SSChigh), early neutrophils (Ly6G+, CXCR2-), late neutrophils (Ly6G+, CXCR2+) and hematopoetic stem-/progenitor cells (LSK cells, Lineage-, Sca-1+, c-Kit+).

##### Photoconversion KikGR

Mice were anesthetized with 1.5–2% isoflurane (vol/vol), delivered in medicine air, and maintained at 37 °C throughout the procedure. Briefly, a skin midline incision was made on the head of the mouse, and the skull was exposed. Photoconversion was performed with a defocused (5-mm beam diameter) violet laser source (405 nm, peak power 4.5 mW, ThorLabs) placed 5 cm above the skull of the brain ischemic region (ipsilateral) for 3 min. Mouse skin was then sutured and allowed to recover on a heating pad until responsive. One hour or six hours after photonconversion, mice were anesthetized with isofluorane and transcardially perfused with 20 ml cold PBS containing Heparin (2U/ml). Cell suspensions from brain and skull (ipsilateral and contralateral), spleen and femur were isolated as indicated below for flow cytometric analysis and the percentage of photoconverted red cells (KikGR+) was analyzed in the appropriate cell populations.

##### Cell isolation

Mice were deeply anaesthetized with ketamine (120mg/kg) and xylazine (16mg/kg) and transcardiacally perfused with cold PBS. The skull, femur, spleen and brain were immediately harvested and kept on ice. The olfactory bulb and cerebellum were discarded, and the brain was mechanically dissociated in RPMI media with a douncer homogenizer, followed by a Percoll gradient centrifugation. For the isolation of skull bone marrow, meninges were peeled from the skull cup under the microscope and not included in this study. The isolated calvarium was cut into small pieces and mechanically dissociated on top of a 40 μm cell strainer with the end of a 1-mL syringe plunger. After centrifugation at 500g for 7 minutes, cell suspensions were washed with PBS or FACS buffer.

##### Flow cytometry of KikGR animals

For differentiation of live and dead cells we stained cells with the Zombie NIR (BioLegend). For surface marker analysis, nonspecific binding was blocked by incubation for 10 min at 4 °C with anti-CD16/CD32 antibody (Biolegend, 5 ng/μL) antibody and stained with the appropriate antibodies for 15 min at 4 °C. The following antibodies were used for extracellular staining: CD45 (clone 30-F11, eFluor450, Invitrogen # 48-0451-82), CD11b (clone M1/70, PE/Cy7, Invitrogen # 25-0112-82), Ly6C (clone HK1.4, PerCP/Cy5.5, BioLegend #128012), Ly6G (clone 1A8-Ly6g, PE-eFluor610, Invitrogen #61-9668-82), CD3 (Clone 17A2, APC, Invitrogen # 17-0032-82) and CD19 (eBio1D3, APC/eFluor780, Invitrogen # 47-0193-82).

Cells were washed with FACS buffer, resuspended in 200 μl of FACS buffer and acquired using a Cytek® Northern Lights (Cytek® Biosciences, US) and analyzed using FlowJo software (Treestar, US). Isotype controls were used to establish compensation and gating parameters.

#### Multiplexed RNAscope smFISH

Large tissue section staining and fluorescent imaging were conducted largely as described previously.^96^ Sections were cut from fixed frozen samples embedded in OCT at a thickness of 10 μm using a cryostat, placed onto Hydrophilic Plus slides (BioSB) and stored at -80°C until stained.

Fixed frozen tissue sections were processed using a Leica BOND RX to automate staining with the RNAscope Multiplex Fluorescent Reagent Kit v2 Assay (Advanced Cell Diagnostics, Bio-Techne), according to the manufacturers’ instructions. Probes may be found in Table S1, tab 37. Prior to staining, sections were post-fixed in 4% paraformaldehyde in PBS at 4°C for 15 minutes, then dehydrated through a series of 50%, 70%, 100%, and 100% ethanol, for 5 minutes each. To maximize adhesion of sections, slides were then baked at 37°C for 30 minutes. Following manual pre-treatment, automated processing included digestion with Protease III for 10 minutes prior to probe hybridisation. Tyramide signal amplification with TSA Vivid 520, TSA Vivid 570, and TSA Vivid 650 (Tocris Bioscience) and TSA-biotin (TSA Plus Biotin Kit, Perkin Elmer) and streptavidin-conjugated Atto 425 (Sigma Aldrich) was used to develop RNAscope probe channels.

To reduce autofluorescence, slides were treated immediately post-staining with TrueBlack® Plus Lipofuscin Autofluorescence Quencher (Biotium) for 5 minutes, then washed several times with PBS before mounting with ProLong Gold Antifade Mountant (Thermo).

#### High-resolution imaging

Stained sections were imaged with a Perkin Elmer Opera Phenix Plus High-Content Screening System, in confocal mode with 2 μm z-step size, using a 40X (NA 1.1, 0.149 μm/pixel) water-immersion objective. Channels: DAPI (excitation 375 nm, emission 435-480 nm), Atto 425 (ex. 425 nm, em. 463-501 nm), TSA Vivid 520 (ex. 488 nm, em. 500-550 nm), TSA Vivid 570 (ex. 561 nm, em. 570-630 nm), TSA Vivid 650 (ex. 640 nm, em. 650-760 nm).

#### Image stitching

Confocal image stacks were stitched as two-dimensional maximum intensity projections using proprietary Acapella scripts provided by Perkin Elmer. Resulting images were viewed as OME-TIFFs using OMERO Plus (Glencoe Software).

#### Scanning electron microscopy

Human skull samples were freshly prepared, dissected and immersed into fixative (4% PFA and 2.5% glutaraldehyde in 0.1 M sodium cacodylate buffer, pH 7.4; Science Services). After decalcification in EDTA for 1 month at 4°C and 1 month at room temperature, skull samples were washed, further dissected into 1x2 mm slabs bearing dura and bone layers and immersion fixed for 24h in fixative. We applied a standard rOTO en bloc staining protocol including postfixation in 2% osmium tetroxide (EMS), 1.5% potassium ferricyanide (Sigma) in 0.1 M sodium cacodylate (Science Services) buffer (pH 7.4).^97^ Staining was enhanced by reaction with 1% thiocarbohydrazide (Sigma) for 45 min at 40°C. The tissue was washed in water and incubated in 2% aqueous osmium tetroxide, washed and further contrasted by overnight incubation in 1% aqueous uranyl acetate at 4°C and 2 h at 50°C. Samples were dehydrated in an ascending ethanol series and infiltration with LX112 (LADD). Blocks were screened for tunnel structures transversing from the bone to the dura on cross sections using sequential trimming (TRIM2, Leica) and light microscopy of semithin sections. Serial sections were taken with a 35° ultra-diamond knife (Diatome) on an ATUMtome (Powertome, RMC) at a nominal cutting thickness of 200 nm and collected on freshly plasma-treated (custom-built, based on Pelco easiGlow, adopted from M. Terasaki, U. Connecticut, CT), carbon coated Kapton tape (kindly provided by Jeff Lichtman and Richard Schalek). Tape stripes were assembled onto adhesive carbon tape (Science Services) attached to 4-inch silicon wafers (Siegert Wafer) and grounded by adhesive carbon tape strips (Science Services). EM micrographs were acquired on a Crossbeam Gemini 340 SEM (Zeiss) with a four-quadrant backscatter detector at 8 kV using ATLAS5 Array Tomography (Fibics). We acquired medium resolution (40-100 nm) images of the entire section and the region of interest and processed in Fiji.^82^

#### Proteomics Sample Preparation

Sample preparation for proteomics analysis was performed as described previously with slight modifications.^98^ Briefly, for mouse samples, SDC lysis buffer (2% SDC, 100 mM Tris-HCl pH 8.5) was used to lyse the cell pellets at 95°C for 45 min at 600 rpm in a thermoshaker. For human samples which were fixed in PFA, prior to the SDC lysis buffer step, the samples were first resuspended in 6% SDS buffer, heat denatured, sonicated and then precipitated using 80% acetone overnight in -20°C. Next day, these samples were centrifuged and the pellet was resuspended in SDC lysis buffer. After this, the procedure remains the same for both mouse and human samples. Naïve meninges samples from mice were lost during sample preparation. The samples in SDC buffer were sonicated in high mode for 15 cycles (30 sec OFF, 30 sec ON) (Bioruptor® Plus; Diagenode). The samples were again heated at 95°C for 45 min at 600 rpm in a thermoshaker. The extracted and solubilized protein concentration was estimated by BCA method and 25 μg of protein was further reduced and alkylated using a final concentrations of 10 mM TCEP and 40 mM CAA in dark, at 45°C for 10 min with 600 rpm in a thermoshaker. The protein samples were digested overnight with Trypsin and LysC (1:50, protease:protein ratio) at 37°C, 1,000 rpm shake. Resulting peptides were acidified with 1% TFA 99% isopropanol with 1:1 volume-to-volume ratio, vortexed and centrifuged to pellet residual particles. The supernatant was transferred to fresh tubes and subjected to in-house built StageTip clean-up consisted of three layers of styrene divinylbenzene reversed-phase sulfonate (SDB-RPS; 3 M Empore) membranes. Peptides were loaded on the activated (100% ACN, 1% TFA in 30% Methanol, 0.2% TFA, respectively) StageTips, run through the SDB-RPS membranes, and washed by EtOAc including 1% TFA, isopropanol including 1% TFA, and 0.2% TFA, respectively. Peptides were then eluted from the membranes via 60 μL elution buffer (80% ACN, 1.25% NH4OH) and dried using vacuum centrifuge (40 min at 45°C). Finally, peptides were reconstituted in 10 μL of loading buffer (2% ACN, 0.1% TFA) and peptide concentration was estimated using PierceTM Quantitative Colorimetric Peptide Assay.

#### Liquid chromatography and mass spectrometry (LC-MS/MS)

The mass spectrometry data was acquired in data independent acquisition (DIA) mode. The LC-MS/MS analysis was carried out using EASY nanoLC 1200 (Thermo Fisher Scientific) coupled with trapped ion mobility spectrometry quadrupole time-of-flight single cell proteomics mass spectrometer (timsTOF SCP, Bruker Daltonik GmbH, Germany) via a CaptiveSpray nano-electrospray ion source. Peptides (50 ng) were loaded onto a 25 cm Aurora Series UHPLC column with CaptiveSpray insert (75 μm ID, 1.6 μm C18) at 50°C and separated using a 50 min gradient (5-20% buffer B in 30 min, 20-29% buffer B in 9 min, 29-45% in 6 min, 45-95% in 5 min, wash with 95% buffer B for 5 min, 95-5% buffer B in 5 min) at a flow rate of 300 nL/min. Buffer A and B were water with 0.1 vol% formic acid and 80:20:0.1 vol% ACN:water:formic acid, respectively. MS data were acquired in single-shot library-free DIA mode and the timsTOF SCP was operated in DIA/parallel accumulation serial fragmentation (PASEF) using the high sensitivity detection-low sample amount mode. The ion accumulation and ramp time was set to 100 ms each to achieve nearly 100% duty cycle. The collision energy was ramped linearly as a function of the mobility from 59 eV at 1/K0 = 1.6 Vs cm−2 to 20 eV at 1/K0 = 0.6 Vs cm−2. The isolation windows were defined as 24 X 25 Th from m/z 400 to 1000.

#### Proteomics data processing

diaPASEF raw files were searched against the human uniport database using DIA-NN.^99^ Peptides length range from seven amino acids were considered for the search including N-terminal acetylation. Oxidation of methionine was set as a variable modification and cysteine carbamidomethylation as fixed modification. Enzyme specificity was set to Trypsin/P with 2 missed cleavages. The FASTA digest for library-free search was enabled for predicting the library generation. The FDR was set to 1% at precursor and global protein level. Match-between-runs (MBR) feature was enabled and quantification mode was set to “Robust LC (high precision)”. The Protein Group column in DIA-NN’s report was used to identify the protein group and PG.MaxLFQ was used to calculate the differential expression.

#### Small animal PET/MRI acquisition

All rodent PET procedures followed an established standardized protocol for radiochemistry, acquisition times and post-processing,^100^^,^^101^ which was transferred to a novel PET/MRI system.^102^ All mice were scanned with a 3T Mediso nanoScan PET/MR scanner (Mediso Ltd, Hungary) with a triple-mouse imaging chamber. A 15-minute anatomical T1 MR scan was performed at 45 min after [18F]-GE180 injection (head receive coil, matrix size 96 × 96 × 22, voxel size 0.24 × 0.24 × 0.80 mm³, repetition time 677 ms, echo time 28.56 ms, flip angle 90°). Injected dose was 13 ± 2 MBq delivered in 200 μl saline via venous injection. PET emission was recorded at 60-90 min p.i. PET list-mode data within 400–600 keV energy window were reconstructed using a 3D iterative algorithm (Tera-Tomo 3D, Mediso Ltd, Hungary) with the following parameters: matrix size 55 × 62 × 187 mm³, voxel size 0.3 × 0.3 × 0.3 mm³, 8 iterations, 6 subsets. Decay, random, and attenuation correction were applied. The T1 image was used to create a body-air material map for the attenuation correction.

#### Human TSPO-PET imaging acquisition

All participants were scanned at the Department of Nuclear Medicine, LMU Munich, using a Biograph 64 PET/CT scanner (Siemens, Erlangen, Germany). Before each PET acquisition, a low-dose CT scan was performed for attenuation correction. Emission data of TSPO-PET were acquired from 60 to 80 minutes^62^^,^^103^ after the injection of 187 ± 11 MBq [^18^F]GE-180 as an intravenous bolus, with some patients receiving dynamic PET imaging over 90 minutes. The specific activity was >1500 GBq/μmol at the end of radiosynthesis, and the injected mass was 0.13 ± 0.05 nmol. All participants provided written informed consent before the PET scans. Images were consistently reconstructed using a 3-dimensional ordered subsets expectation maximization algorithm (16 iterations, 4 subsets, 4 mm gaussian filter) with a matrix size of 336 × 336 × 109, and a voxel size of 1.018 × 1.018 × 2.027 mm. Standard corrections for attenuation, scatter, decay, and random counts were applied. For the AD cohort, emission data of Aβ-PET were acquired from 90 to 110 minutes after injection of 188 ± 10 MBq [^18^F]flutemetamol. Aβ-PET was assessed by a visual read (one expert reader), and the decision of Aβ-positivity/negativity was supported by a software-driven approach implemented in HERMES Gold (V4.17, HERMES Medical Solutions AB, Stockholm, Sweden). One positive evaluated target region (frontal, temporal, parietal, posterior cingulate) defined the scan as positive.

### Quantification and statistical analysis

#### Single-cell RNA data analysis

##### Count matrix generation

Count matrices were created using CellRanger (v. 3.0.2) aligning reads to the mouse genome mm10 (ensrel97). Spliced and unspliced matrices for RNA-velocity^43^ analysis were computed using the velocyto (0.17.17) pipeline. (n=3 pooled animals for sham and n=6 pooled animals for MCAo).

##### Quality control

Samples were jointly analyzed using scanpy^83^ (v. 1.6) and anndata (v. 0.7.5) in Python 3.7. Different quality control filters^104^ were used to account for the characteristics of the different samples: In bone samples, all cells with a mitochondrial read fraction higher than 0.2 were removed. In meninges and brain samples, thresholds were 0.3 and 0.6, respectively. Further, cells with less than 1000 UMI counts (bone samples) and 500 UMI counts (meninges, brain), and more than 50,000 UMI counts were removed. We did not apply a minimum gene filter per cell to retain erythroblasts. All genes expressed in less than 10 cells were removed. To estimate doublets, we used the tool scrublet with a doublet score threshold of 0.1 and removed cells with a higher doublet score. Additionally, we filtered out two small clusters that showed dendritic cell markers as well as markers from other cell types as they might also be doublets, and a sub cluster of early monocytes that showed an increased mitochondrial fraction. Finally, our filtered dataset contained 147,082 cells expressing 17,040 genes coming from 32 samples.

##### Data preprocessing

To normalize the data with scran,^105^ size factors were determined as follows: data were first temporarily normalized by total with a target sum of 10,000 per cell followed by log+1-scaling. Then, for each cell, 30 nearest neighbors were computed and data were clustered with Leiden clustering at default resolution 1. Small clusters with less than 100 cells were merged with closely related clusters based on the PAGA graph. For PAGA graph calculations we used scanpy's implementation with default parameters.^106^ Then, size factors were computed on these clusters and the UMI count data were divided by scran size factors for each cell and log+1-scaled. Then, mitochondrial reads were removed and 4,000 highly variable genes per sample were computed (highly_variable_genes with flavor "cell_ranger" in scanpy). Further, cell cycle scores were computed (score_genes_cell_cycle in scanpy). To evaluate batch effects, PC regression scores for the variance explained by cell cycle, anatomic region and condition were computed for the full dataset and the MCAo replicates, respectively. PC regression scores were lowest in the condition and replicate covariate, respectively, and therefore no batch effect correction was performed.

##### Dendrograms

With scanpy's dendrogram function SciPy’s hierarchical linkage clustering was calculated on a Pearson correlation matrix over regions which was calculated for 50 averaged principal components.

##### Cell type annotations

Cell types were annotated according to a two-step procedure. In a first step a Leiden clustering was calculated on the log-normalized data. The Leiden clusters were annotated with coarse cell type labels according differentially expressed known markers. In the second step leiden clustering with multiple resolutions were calculated for each coarse cell type. Based on differently expressed known markers, as well as additional information like number of genes^107^ and scVelo^43^ implementation of RNA velocity^42^ the clusters were annotated with fine cell types, and coarse annotations were refined.

##### Variance explained by covariates and PC regression

To quantify how strong cell type populations of each region diverge from the other regions the explained variance was calculated by linear regression in PCA space. For each bone the cell type populations were grouped into the given bone vs the other bones. Scores were only calculated if there were at least 20 cells in each of both groups. 50 principal components were calculated for each cell type. A linear regression on the group variable was calculated for each PC component. R2 scores of the linear regression were multiplied by the eigenvalues of the pc components and normalized by the eigenvalue sum, and finally summed up to the variance explained. The significance of each obtained variance explained score was measured via a permutation test. The region annotations were permuted 1000 times. Scores with a p-value below 0.0001 were considered as significant. We decided to exclude scapula in further downstream analysis because we detected an overall decrease in log counts in this sample.

##### Combinatorial DE tests

For each gene, two t-tests were calculated to identify if the gene is upregulated in a group of bones. To define the two bone groups for a given gene, bones were ordered by the gene's mean expression and split in two groups at the highest mean expression gap. The first t-test was conducted on the two groups and the second on the two bones closest to the expression gap. The second test ensures that the expressions of the two closest bones of the two groups are significantly different. The maximal p-value and minimal log fold change of both tests were used to identify DEGs. The chosen thresholds are p < 0.05 and LF change > 1 (> 0.5 for neutrophils analysis).

##### Other differential expression tests

Differences of DAMP expressions and pro- and anti-inflammatory genes were measured with t-tests (p < 0.05, Benjamini-Hochberg correction). The distributions of each DAMP’s expression over CD45+ cells of each bone in the scRNA-seq data were tested for significance differences between conditions. The pro- and anti-inflammatory genes were tested individually as well as the mean expression of pro- and anti-inflammatory gene sets respectively between groups of bones and conditions.

##### Ligand receptor (LR) interactions

For each bone ligand receptor interaction pairs between cell types were calculated with CellPhoneDB's^39^ statistical analysis. An interaction is defined by four variables: ligand, receptor, ligand cell type and receptor cell type. For a fair comparison between bones, pairs were only calculated on cell types that had at least 10 cells in each bone. The statistical analysis was applied on log normalized counts. 400 cells per cell type were sampled to generate a uniform background distribution of the permutation test which otherwise would be skewed towards highly abundant cell types. Cell types with more than 400 cells were down sampled using geometric sketching^108^ (geosketch v 1.2), while the other cell types were up sampled. Strict thresholds based on the CellPhoneDB p-values were applied to reduce the number of false positives: Interactions were considered as significant for p-values equal to 0 (1000 permutations). Interactions were only considered unique to a bone group if the p-value difference between that group and the non-significant group was above 0.95.

##### Gene ontology enrichment

Enrichment of Gene ontology (GO) terms for biological processes were calculated using GProfiler.^109^

##### RNA velocity

RNA velocity^42^ in its scVelo^43^ (v 0.2.3) implementation was used as follows: the dataset with spliced and unspliced raw counts was reduced to the given cell type and condition. Then genes were filtered to 2000 genes with at least 20 counts each, and cells were normalized (filter_and_normalize function in scVelo). First and second order moments for velocity estimation with the scVelo's dynamical model were calculated with default parameters.

##### Pseudotime analysis

Diffusion pseudotime^44^ was calculated to order cells along the neutrophil maturation trajectory. For naive, sham and MCAo a PCA and neighbors’ graph were recalculated on the neutrophils population. The default parameters of scanpy's tl.dpt function was used. As root point we selected the most extreme pro-neutrophil cell from the umap. For cell density visualization along pseudotime^44^ the cell count was smoothed with a Gaussian kernel according to the default parameters of seaborn's (v 0.11.1) kdeplot function. Densities were normalized for each region separately.

##### Donor Deconvolution based on SNPs

To enable statistical tests between different groups in the scRNA-seq data and validate the obtained cell type proportions we deconvolved the samples of pooled animals based on obtained SNPs profiles of the measured transcripts. For SNPs calling we used cellsnp-lite^110^ (v. 1.2.2). Based on the obtained SNPs of each cell vireo^111^ (v. 0.2.3) was used to demultiplex the 3 animals in each pooled sample. Erythrocytes were removed from the analysis as they only express the hemoglobin genes which leads the deconvolution algorithm to identify Erythrocytes as one donor.

##### Correlation of proportions with flow cytometry data

Pearson-correlation between cell type proportions of scRNA-seq and flow cytometry was measured over all cell types obtained in flow cytometry. Since flow cytometry measurements are relative to gated subgroups, we transformed the proportions of scRNA-seq cell types relative to comparable coarse subgroups as well. The significance of proportion differences between bones or conditions were obtained by t-tests over flow cytometry samples and over SNPs based deconvolved animals of the pooled scRNA-seq samples. A few cell type proportion differences were observed consistently with statistical significance (p < 0.05) in both methods. E.g., we observed a significantly higher number of mature B-cells in sham-operated and MCAo operated compared to naïve animals (Table S1, tab 3). Other cell type differences were observed with either one of the methods, but did not always reach statistical significance in both (Table S1, tabs 3 and 4). Upon injury, B-cell progenitors were depleted in both the flow cytometry and the scRNAseq data. Moreover, mature neutrophils in the calvaria had a strong trend for an increased cell proportion that, however, did not reach significance (Table S1, tabs 1–4).

##### Other differential expression tests

Differences of DAMP expressions and pro- and anti-inflammatory genes were measured with t-tests (p < 0.05, Benjamini-Hochberg correction). The distributions of each DAMP’s expression over CD45+ cells of each bone in the scRNA-seq data were tested for significance differences between conditions. The pro- and anti-inflammatory genes were tested individually as well as the mean expression of pro- and anti-inflammatory gene sets respectively between groups of bones and conditions.

#### Bulk RNA data analysis for 5xFAD dataset

BCL files were demultiplexed with the bcl2fastq software from Illumina. After quality control with FastQC, reads were aligned using rnaSTAR65 to the GRCm38 (mm10) genome with ERCC synthetic RNA added. Read counts were collected using the parameter “quantMode GeneCounts” of rnaSTAR and using the unstranded values. We filtered out data points with less than 6000 genes or a mitochondrial fraction above 0.0015. Data points were log-normalized by total counts. The significance of the *Tspo* difference between WT and 5xFAD was calculated with a t-test. Differentially expressed genes between bones were obtained from t-tests with Benjamini-Hochberg correction and a p-value threshold of 0.05. The PCA was calculated on log normalized counts. (n=3 5xFAD, 5 wildtype animals, 50 cells per sample, 69 samples in total: 37 wildtype, 32 5xFAD).

#### Bulk RNA data analysis for MCAo dataset

For the count matrix generation reads were aligned to the GRCm39 genome with Salmon^112^ using the nf-core/rnaseq pipeline^113^ (v. 3.9). No sample was excluded after quality control. Differentially expressed genes between bones were obtained using the DESeq2 model.^114^ The UMAP was calculated based on a PCA on log-normalized counts (normalization by total counts). For the comparison with scRNA-seq mouse data the pearson-correlation between mean log raw counts over genes of single cell and bulk data of each bone was calculated. (n=5 naïve, 5 sham, 6 MCAo animals).

#### Statistical analysis of KikGR animals

Due to high penetration of UV laser, some Cd11b+ cells, that could be microglia were illuminated as well. To control for this offset, the percentage of microglia illuminated was subtracted from myeloid cells. Acquired data was analyzed and visualized using GraphPad Prism (version 8.0) using two-tailed t-test. (Each dot represents a biological replicate, n=5 for 1h and n=4 animals for 6h, data represented as ± SEM).

#### Image Analysis

##### 2-Photon analysis

We analyzed the 2-photon image stacks as maximum intensity projected time-series (3 frames per batch). We trained a random forest pixel classifier (Ilastik^115^ with default settings) on 3 images of the green channel (LysM-eGFP) and used that for subsequent classification of the LysM-eGFP channel of each image stack. This gave 8-bit probabilities for each frame, which we then thresholded and watershed-segmented using ImageJ.^82^ We performed this procedure with the pixel count (=area) occupied by GFP+ cells. We normalized LysM^+^ cell density at each time point to the cell density at baseline for each ROI. To account for other influences such as laser skull exposure, laser illumination, and anesthesia we further normalized the fold changes in sham and MCAo to those observed in naïve animals at the same time point. The quantification graph was analyzed and visualized using GraphPad Prism (version 8.0) using two-tailed t-test and simple linear regression. (n=3 for naïve and sham and n=5 animals for MCAo, data represented as ± SEM.)

##### Tissue cleared mouse head analysis

For quantification of the mouse heads, manual ROIs were drawn on the frontal and parietal skull bones. C57BL6/J mice has been used for naïve condition quantification. The areas above manually selected threshold based on bone marrow coverage were recorded. The quantification graph was analyzed and visualized using GraphPad Prism (version 8.0) (Ordinary one-way ANOVA with multiple comparisons). (n=3 per group, data represented as ± SEM).

##### *Nr4a1* analysis

Three naïve mice heads and femurs were labeled with *Nr4a1* antibody and propidium iodide (for nuclei staining) and high-resolution images were obtained for 20 ROIs from the mouse skulls and 14 ROIs from the femurs (Figure 2D). We then quantified the number of voxels with a signal above threshold and found that a significantly higher percentage of *Nr4a1* positive voxels in the mouse head (Figure 2E). For the quantification of *Nr4a1*, same-sized regions of interest (ROIs) of 12x scans of the Nr4a1 channel was used. For each of these, an expert manually selected the signal activation threshold after visual inspection in an image analysis software (e.g., Fiji). These thresholds were then used in Python to obtain binary masks of active expression of the same size as the ROI. Pixels that have higher intensity than the threshold in the Nr4a1 channel are assigned the value of 1 (positive pixels), and the rest, 0. The total amount of pixels above threshold in each ROI is the number of positive pixels in the binary mask, whereas the percentage of signal in the volume is calculated as the number of positive pixels divided by the number of total pixels in the ROI. The quantification was analyzed and visualized using GraphPad Prism (version 8.0) using two tailed t-test. (n=3 per group, data represented as ± SEM).

##### Human skull segmentation and channel measurement

Segmentation of the skull channels network and measurement of skull meninges channels were performed by using syGlass (https://www.syglass.io/). This software allows to visualize the microscopic data of the light sheet microscopes in 3D in Virtual Reality (VR). To segment and measure the data, smaller ROIs were cut out in VR. In these ROIs the openings in the meninges connected to the skull meninges channels were segmented up to the channel network that connects to the first bone marrow chambers in the skull. Then the Virtual reality software was used to change visual settings in order to measure, segment and to generate videos in 3D. The quantification was analyzed and visualized using GraphPad Prism (version 8.0) using two tailed t-test. (n=7 post-mortem samples, 23 ROI in total, 522 channels, data represented as ± SEM).

##### Graph Representation of the Skull Channels

In order to achieve an additional compact representation of the skull channels we extracted a graph representation from the human skull segmentation described above using VR. We used the *voreen* open-source software to generate a centerline representation and second a graph representation with edges and nodes. In total, the skull channel graph consists of 399 nodes, and 440 edges with an average node degree of 2.21. The images are rendered using the Syglass software (Figure S6R).

#### Proteomics data analysis

Both human and mouse samples were jointly analyzed using scanpy (v. 1.9.1) and anndata (v. 0.7.6) in Python 3.8 and follows similar analysis pipeline. (n=3 independent samples each for bones and brain for all conditions, n=3 for meninges MCAo and sham conditions.)

##### Quality control

All proteins expressed in less than half of the bone samples were filtered out. For mice, the meninges were excluded from the filtration criterion since we identify the least number of proteins.

##### Data preprocessing

The data was further log-transformed and normalised per sample. KNN imputation was our method of choice using KNNImputer (n_neighbors=5) from the sklearn package (v. 0.22).

##### Gene set enrichment analysis (GSEA)

Enrichment of Gene ontology (GO) terms for biological processes were analysed using GProfiler^109^ and Enrichr.^116^

##### Weighted correlation network analysis (WGCNA)

To identify the different modules of correlated genes in our datasets, WGCNA were used [python version: PyWGCNA^117^]. This method is an unsupervised algorithms for finding clusters (modules) of highly correlated genes based on a graph where nodes represents genes/proteins and the adjacency matrix is calculated based on the co-expression similarity between the nodes. Modules are then identified as clustered of interconnected nodes (genes/proteins) using hierarchical clustering. Gene ontology enrichment is further applied for genes/proteins identified from each module are to determine the biological processes pathways related to these modules.

##### Dendrograms

With scanpy’s dendrogram function scipy’s hierarchical linkage clustering was calculated on a Pearson correlation matrix over regions which was calculated for 50 averaged principal components.

##### Differential expression tests

To identify differentially regulated genes across two conditions (e.g. one bone vs the rest), scanpy’s method that ranks genes group using t-tests was used. The maximal p-value and minimal log fold change were used to identify differentially expressed proteins/genes (DEPs). The chosen thresholds are p < 0.05 and LF change > 1. These DEPs were further used to plot the volcano plots.

#### Small animal PET/MRI analysis

To capture skull specific PET signal from the skull in three wildtype mice, immediately after in vivo TSPO-PET imaging of mice, the brain, blood (perfusion via PBS) and all tissue surrounding the skull bone was removed. The skull bone of each mouse was imaged via a second TSPO-PET session. The signal attributable to the skull in the in vivo TSPO-PET images were compared to the signal in the respective skull-only TSPO-PET as standard of truth. For this purpose, an in-house CT template to delineate the skull bone in PET was used and a cluster-based analysis (k-means clustering) was performed, dividing the skull into 50 regions of increasing PET signal intensity. We studied TSPO-PET images of 5xFAD mice (n=6) and wild-type mice (n=6), all female at an age of 4.5 months. Normalization of injected activity was performed by cerebellar scaling^118^ to ensure consistency with human data. TSPO labeling in the skull was obtained in each mouse from a fronto-parietal volume-of-interest (comprising 24 mm³) and from a temporal volume-of-interest (comprising 16 mm³), which were semi-automatically delineated using an in-house CT template. Fronto-polar skull was spared to avoid signal spill-over from regions with strong amyloidosis and microglial activation inside the brain. TSPO labeling of the skull was compared 5xFAD and wild-type mice. Voxel-wise differences were calculated to allow a volume-of-interest independent validation of elevated skull tracer binding in 5xFAD mice. The quantification and visualization was done using GraphPad Prism (version 8.0) using two-tailed t-test (data represented as ± SEM) and correlation analysis.

#### Human TSPO-PET imaging analysis

All TSPO-PET data were analyzed using PMOD. Spatial normalization was performed to a tracer specific templates in the Montreal Neurology Institute (MNI) space which was acquired via MRI-based spatial normalization. All images were normalized by cerebellar grey matter scaling (defined by the Hammers atlas^119^) prior to analysis and a standardized-uptake-value (SUV) analysis served for pseudo-reference tissue independent validation.

For stroke, multiple sclerosis and 4R tauopathy patients we defined three target regions based on a voxel-wise exploratory analysis: temporopolar skull (comprising 18 cm³), skull base (comprising 97 cm³), and prefrontal skull (comprising 7 cm³). All regions were semi-automatically delineated using the human CT template available in PMOD. Region-based PET values were normalized to a composition of values of exactly age-matched (≤ 1 year difference) controls at the group level. Voxel-wise differences (% vs. age-matched controls) were calculated to allow a volume-of-interest independent validation of elevated skull tracer binding in all patient groups. Following the region-based approach, we used compositions of exactly age-matched controls for this calculation.

For the AD cohort, TSPO labeling in the calvaria was obtained in each participant from a large fronto-parietal volume-of-interest (comprising 66 cm³), which was semi-automatically delineated using the human CT template available in PMOD. Posterior and frontal calvaria was spared to avoid signal spill-over from sinuses and extracranial structures. Furthermore, we used a Brainnetome^66^ atlas-based classification of cortical brain regions and corresponding calvaria regions to test for regional calvaria-brain associations. To this end, we increased the dimension of the atlas by a factor of 1.2 and we delineated all volumes-of-interest that were represented in the calvaria as defined by the CT template (≥50% of voxels included). This approach resulted in 64 individual calvaria-brain region pairs. TSPO labeling of the calvaria was compared between AD patients with β-amyloid pathophysiology (AD) and β-amyloid negative controls. Voxel-wise differences were calculated to allow a volume-of-interest independent validation of elevated calvaria tracer binding in patients with AD. TSPO labeling of the calvaria was correlated with age, sex, and cognitive testing (MMSE, CERAD, CDR) as well as with β-amyloid levels in CSF. Calvaria-brain associations of TSPO-PET were tested for the global calvaria volume-of interest with Braak stage and β-amyloid related composite brain regions. Furthermore, calvaria-brain associations were tested by a correlation matrix of the predefined 64 volume-of-interest pairs. Single region increases in patients with AD vs. healthy controls were correlated between calvaria and brain regions.

As a validation of specificity, we performed an additional analysis of TSPO tracer uptake in the corpus vertebrae of C2. This bone was chosen as a negative control region since it was captured in nearly all acquisitions as the most remote bone structure relative to skull. The analysis was performed manually using HERMES Full Flex (V4.17, HERMES Medical Solutions AB, Stockholm, Sweden). A 1.0 mm3 sphere was placed in the center of C2 and SUV was extracted and normalized to cerebellar uptake (i.e. SUVr).

For longitudinal imaging, individual follow-up TSPO-PET SUVr of the fronto-parietal region were compared to baseline by a paired t-test for both patients of the AD continuum and controls. For patients with stroke, we used SUV normalization since distinct changes of tracer uptake in whole brain did not allow reference tissue normalization for the longitudinal analysis. Here, individual follow-up TSPO-PET SUV of the infarct region were compared to baseline by a paired t-test, as no 3 month follow-up scans were available for healthy controls. As a region independent analysis we used the skull template implemented in SPM (V12, University College of London, London, UK) running in Matlab version R2016 (MathWorks Inc., Natick, MA) and performed a voxel wise paired t-test analysis between baseline and follow-up images of patients of the AD continuum and controls (SUVr) as well as patients with stroke (SUV). A p value threshold of 0.05, incorrectly for multiple comparisons, was considered significant to obtain a pattern of changes rather than only peak clusters with the highest changes. Significant changes were displayed as a skull surface projection. The ActiGliA cohort also allowed to correlate changes of TSPO tracer uptake in brain with changes of TSPO tracer uptake in skull for patients of the AD continuum and controls (not feasible in stroke due to the individual locations of the lesion). Skull was treated as one region of interest as described for the longitudinal analysis above. Brain was parcellated into 246 regions of the brainnetome atlas.^120^ Chances of all 246 brain regions were correlated with changes in skull separately for patients of the AD continuum and controls. An FDR correction for multiple comparisons was applied to the respective p values.

#### Statistics for human TSPO-PET imaging

Group comparisons of VOI-based PET results between patient groups with mixed neurological disorders and controls (n=5 groups) were assessed by 1-way ANOVA and Bonferroni post hoc correction for multiple comparisons using IBM SPSS Statistics (version 22.0; SPSS). All data were controlled for age, sex and the TSPO single nucleotide polymorphism at the individual subject level.

Group comparison of Human TSPO-PET results between controls and AD patients were assessed by a two-tailed t-test in SPSS Statistics (version 22.0; SPSS), controlled for age, sex and the TSPO single nucleotide polymorphism. For correlation analyses, Pearson coefficients of correlation (R) were calculated. A threshold of P less than 0.05 was considered to be significant for the rejection of the null hypothesis. The visualization of the data was done using GraphPad Prism (version 8.0), (data represented as ± SEM).

### Additional resources

Videos related to this work: https://www.discotechnologies.org/Calvaria/.

## Data Availability

• Single-cell RNA sequencing data raw counts, matrices and annotation and bulk RNA datasets are available via NCBI’s GEO (GSE192616), proteomic data is available on PRIDE, accession code: PXD041665 and patient source file human TSPO-PET imaging study can be found under supplemental information. Imaging data is available upon request from the corresponding author. • All code used in this study can be found as jupyter notebooks in the project github repository: https://github.com/erturklab/skull_immune. • Any additional information required to reanalyze the data reported in this paper is available from the lead contact upon request.

## Supplemental information

Table S1. All relevant tables relating to RNA-seq analysis, related to Figures 1 and 2

Table S2. All relevant tables relating to proteomics analysis, related to Figures 3 and 5

Table S3. The source file for TSPO-PET imaging data (patients and mice), related to Figures 6 and 7
